# Target delivery of doxorubicin tethered with PVP stabilized gold nanoparticles for effective treatment of lung cancer

**DOI:** 10.1038/s41598-018-22172-5

**Published:** 2018-02-28

**Authors:** Vaikundamoorthy Ramalingam, Krishnamoorthy Varunkumar, Vilwanathan Ravikumar, Rajendran Rajaram

**Affiliations:** 1 DNA Barcoding and Marine Genomics lab, Department of Marine Science, Bharathidasan University, Tiruchirappalli, 620 024 Tamil Nadu India; 2 Cancer Biology Lab, Department of Biochemistry, Bharathidasan University, Tiruchirappalli, 620 024 Tamil Nadu India

## Abstract

Development of drug delivery system conjugated with doxorubicin (dox) on the surface of AuNPs with polyvinylpyrrolidone (Dox@PVP-AuNPs), we have demonstrated that human lung cancer cells can significantly overcome by the combination of highly effective cellular entry and responsive intracellular release of doxorubicin from Dox@PVP-AuNPs complex. Previously drug release from doxorubicin-conjugated AuNPs was confirmed by the recovered fluorescence of doxorubicin from quenching due to the nanosurface energy transfer between doxorubicinyl groups and AuNPs. Dox@PVP-AuNPs achieved enhanced inhibition of lung cancer cells growth than free Doxorubicin and PVP-AuNPs. The *in vitro* cytotoxic effect of PVP-AuNPs, free Dox and Dox@PVP-AuNPs inhibited the proliferation of human lung cancer cells with IC_50_ concentration. Compared with control cells, PVP-AuNPs and free Dox, Dox@PVP-AuNPs can increases ROS generation, sensitize mitochondrial membrane potential and induces both early and late apoptosis in lung cancer cells. Moreover, Dox@PVP-AuNPs highly upregulates the expression of tumor suppressor genes than free Dox and PVP-AuNPs and induces intrinsic apoptosis in lung cancer cells. From the results, Dox@PVP-AuNPs can be considered as an potential drug delivery system for effective treatment of human lung cancer.

## Introduction

Lung cancer is the leading cause of cancer related death among men in worldwide and is the second among women, with a 5-year survival rate is only 18%^1^. Lung cancers are classified as small cell lung cancer (13%) and non-small cell lung cancer (NSCLC) (87%) according to the purposes of treatment^2^. Surgical resection remains the mainstay of treatment for early-stage NSCLC. Unfortunately, the majority of lung cancers are diagnosed at an advanced stage. For advanced NSCLC, the platinum-based regimen is the present standard first-line chemotherapy^3^. However, the response rate to chemotherapy was less than 30%. What’s worse, many patients suffered serious side effect after chemotherapy. Target therapy, especially the use of tyrosine kinase inhibitors (TKIs) has improved the outcome of those patients. But, TKIs just benefit for the patients with EGFR mutation^4^, ranging from ~15% in Caucasians to ~50%^5^, and 95% of them are adenocarcinomas^6^. Thus, to further explore valuable diagnostic and novel therapeutic targets is in urgent need.

Application of nanotechnology in medicine is foreseen guide us to act against the preceding problems^7^. Basically, nanoparticles can be defines as ultra-dispersed and solid supramolecular structures with nanometre in size ranging from 10–100 nm. Among the various metal nanoparticles used for biomedical applications, the gold nanoparticles (AuNPs) attracted significant interest due to its chronological applications in art and ancient medicine and improved biomedical applications^8,9^. Recently many reports have been demonstrated that AuNPs freely permeate blood vessels and tissue into cancer foci and authenticating that AuNPs has effective drug carrier with the application of reducing cytotoxicity to neighbouring cells^10^.

In biomedical applications, AuNPs have become a prospective application for the development of drug delivery systems^11^. There are numerous chemotherapeutic agents comprising camptothecin, taxenes, platinating agents and nucleoside and nucleotide analogs have been used against certain cancer types for last few decades^12^. Though, these chemotherapeutic agents have some demerits by causing both cancer and normal cells and also associated with secondary responses including cardiotoxicity, cytotoxicity, neurotoxicity, nephrotoxicity and ototoxicity^13^. In modern research, Doxorubicin is a front line anticancer drug often conjugated with nanoparticles for drug delivery has been used. Doxorubicin can be easily dissolved, entangled, conjugated or attached with nanoparticle matrix and enhance the anticancer efficacy of chemotherapeutic agents and also reduce side effects in cancer treatment^14^.

The development of multidrug resistance to chemotherapy remains a major challenge in the treatment of cancer. Resistance exists against every effective anticancer drug and can develop by numerous mechanisms including decreased drug uptake, increased drug efflux, activation of detoxifying systems, activation of DNA repair mechanisms, evasion of drug-induced apoptosis, etc^15^. In the present study, we aimed to synthesis the PVP stabilized AuNPs conjugated with Doxorubicin (Dox@PVP-AuNPs) for effective treatment of A549, H460 and H520 human lung cancer cells. The physicochemical properties of Dox@PVP-AuNPs such as average particle size, zeta potential and *in vitro* drug release were investigated. We also demonstrated the effect of Dox@PVP-AuNPs in the expression of p53 and its upstream targets in the p53-dependent intrinsic apoptotic pathway in human lung cancer.

## Results

### Preparation and characterization of PVP-AuNPs

After incubation of 10 min at 70 °C in the magnetic stirrer, a visual color change from yellowish to colourless was observed an addition of CTAB into HAuCl_4_ solution. After mixing of NaBH_4_ with colourless solution, the color changed to dark violet indicating the formation of AuNPs. It was further confirmed using UV-vis spectroscopy that shows maximum absorbance at 525 nm indicating the presence of AuNPs and absorbance due to surface plasmon resonance (SPR) of AuNPs after 1 hr reaction. As chemically prepared AuNPs were characterized using HRTEM analysis showed that mostly spherical in shape with 13.6 nm (Fig. S1a and b) and size of AuNPs also confirmed from the measurement of the diameter of more number of AuNPs. The histogram of size distribution was obtained (Fig. S1c) and an average diameter of AuNPs was found to be a 12 nm. A zeta potential value of AuNPs prepared using reduction of HAuCl_4_ by NaBH_4_ was found to be an −34.3 mV, a moderate stability. SAED pattern of AuNPs (Fig. S1d) confirmed the presence of Au element and AuNPs was face-centred cubic (fcc) crystal structure with corresponding lattice panels at (1, 1, 1), (2, 0, 0), (2, 2, 0) and (3, 1, 1) planes of Au element and this was confirmed by the JCPDS database (PDF No.: 65–2870).

**Figure 1:**
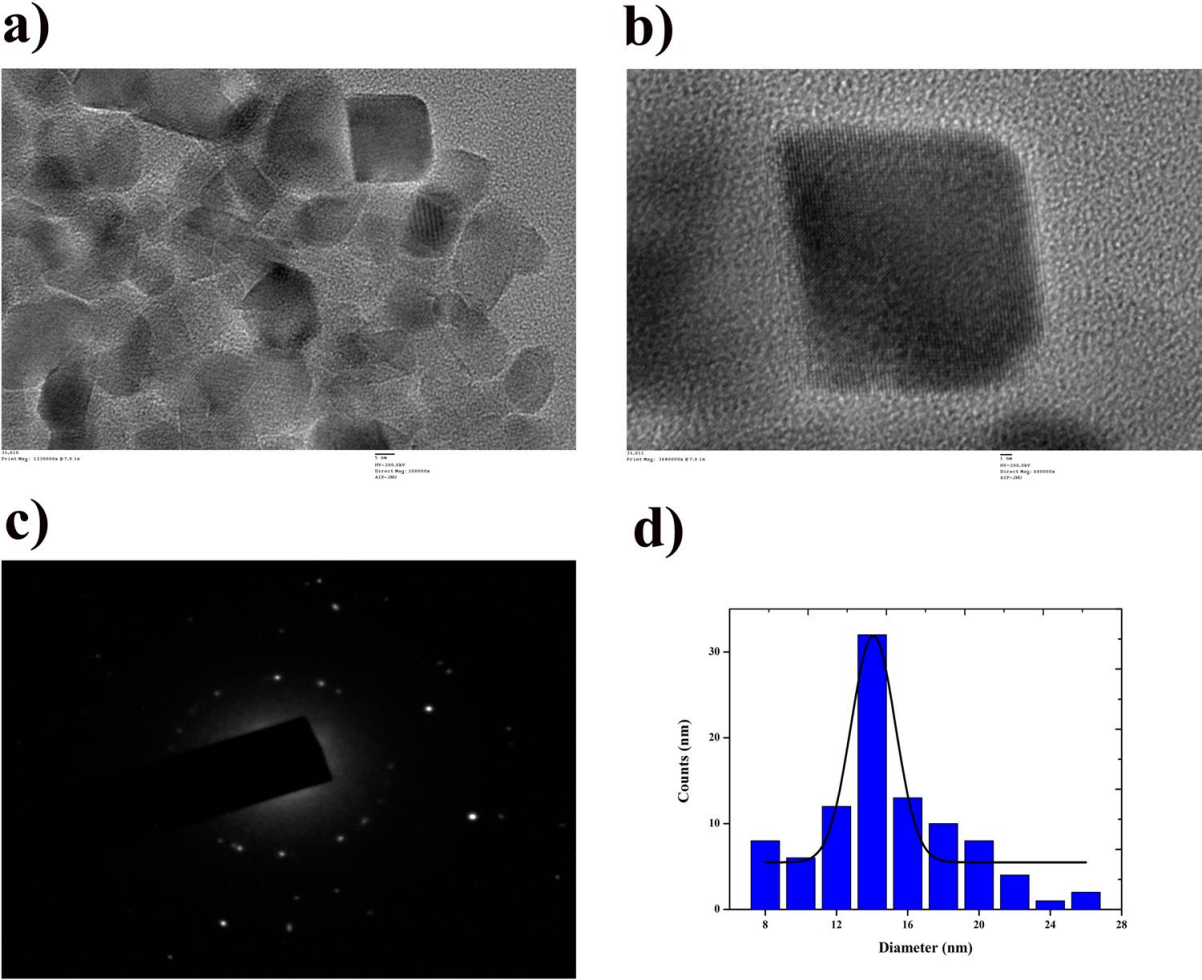
Morphology, size and shape were analysed using TEM micrographs of PVP-AuNPs (**a** and **b**). Crystalline nature of the PVP-AuNPs was analysed using the SAED pattern (**c**) and distribution of PVP-AuNPs according to its size and the average size of the AuNPs is 14 nm (**d**).

As prepared AuNPs was stabilized using PVP by the dynamic stirring method and it further characterized using HRTEM, zeta potential, SAED and NMR. HRTEM (Fig. 1a) illustrates the coating of PVP on surface of AuNPs and single particle of AuNPs (Fig. 1b) clearly illustrates the PVP stabilization on entire surface of AuNPs. Zeta potential of AuNPs was measured to confirm the successful coating with PVP and the results indicated that the stability of AuNPs after stabilized with PVP was increased from −34.3 mV to −18.7 mV due to organic layer over the surface of AuNPs. The addition of PVP to AuNPs reduce the stability as well as the size of AuNPs, HRTEM analysis showed that the size of AuNPs after coated with PVP was 14.5 nm (Fig. 1c). SAED pattern of PVP-AuNPs powder shows the evidence for some characteristics peaks of gold at 2θ value (Fig. 1d). The Bragg’s reflection for PVP-AuNPs was found at (1, 1, 1), (2, 0, 0), (2, 2, 0) and (3, 1, 1) planes of the face-centred cubic (fcc) crystal structure corresponding to the Au element (JCPDS No.: 65–2870).

^1^H and ^13^C NMR spectrum of PVP-AuNPs showed the elemental group for PVP and confirms the coating of PVP around AuNPs. The ^1^H NMR spectrum of PVP-AuNPs showed aromatic groups around δ 7.3, seven methoxy signal at δ 3.240, 3.388, 3.405, 3.672, 3.690 3.730 and 4.814 and an aromatic methyl group at region between δ 1.424 and 2.378 (Fig. S2a). The ^13^C NMR spectrum showed that a carbonyl carbon at 175.5 ppm bearing with double bonded oxygen, three addition aromatic carbons at the region of 76.8, 77.1 and 77.4 ppm and methyl carbon signals were obtained at the region of 18.3 and 31.5 ppm (Fig. S2b).

### Conjugation of Dox@PVP-AuNPs

**Figure 2:**
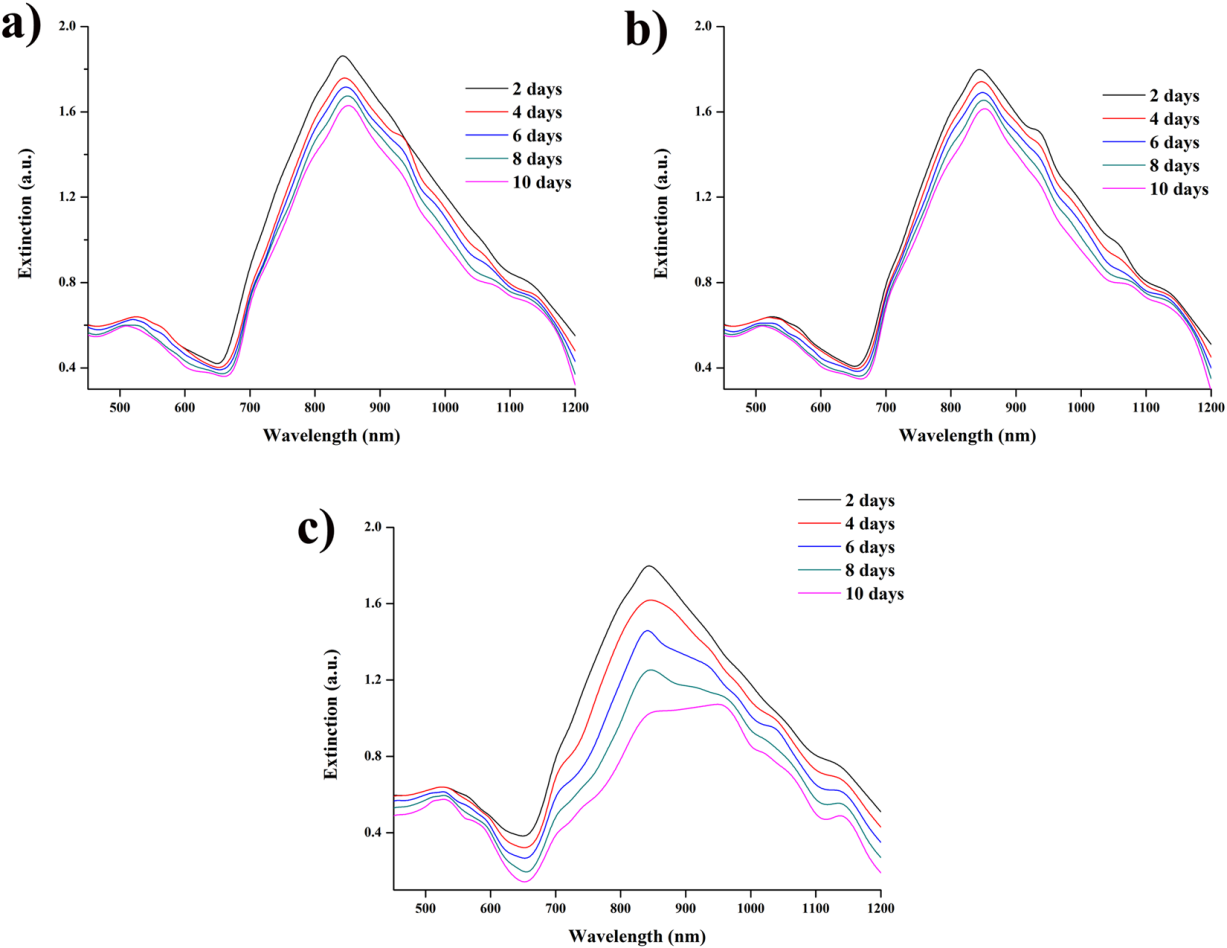
UV- Vis extinction spectra of Dox@PVP-AuNPs at different pH values (**a**) pH 5 (**b**) pH 5.8 (**c**) pH 7.4 for the period of ten days.

The interaction of DOX molecules with PVP-AuNPs was determined by predominant quenching of DOX fluorescence in presence of PVP-AuNPs (Fig. S11). As shown in Fig. S3, the fluorescence intensity of DOX was decreased by increasing the concentration of PVP-AuNPs is due to increasing load of PVP-AuNPs into DOX. As well as, the emission spectrum of AuNPs was decreases on increasing the concentration of Dox is due to increasing load of Dox into PVP-AuNPs. After conjugation of DOX with PVP-AuNPs, the effect of pH on fluorescence intensity was studied by varying pH. At pH 5 and pH 5.8 (acetate buffer), the fluorescent intensity at 585 nm was significantly increased (Fig. 2a and b) with increasing the time interval and fluorescent intensity at 585 nm was significant decreases at pH 7.4 (1× PBS) by increasing the time interval (Fig. 2c) and maximum fluorescence emission was observed after 10 hr incubation.

### *In vitro* release of doxorubicin

**Figure 3:**
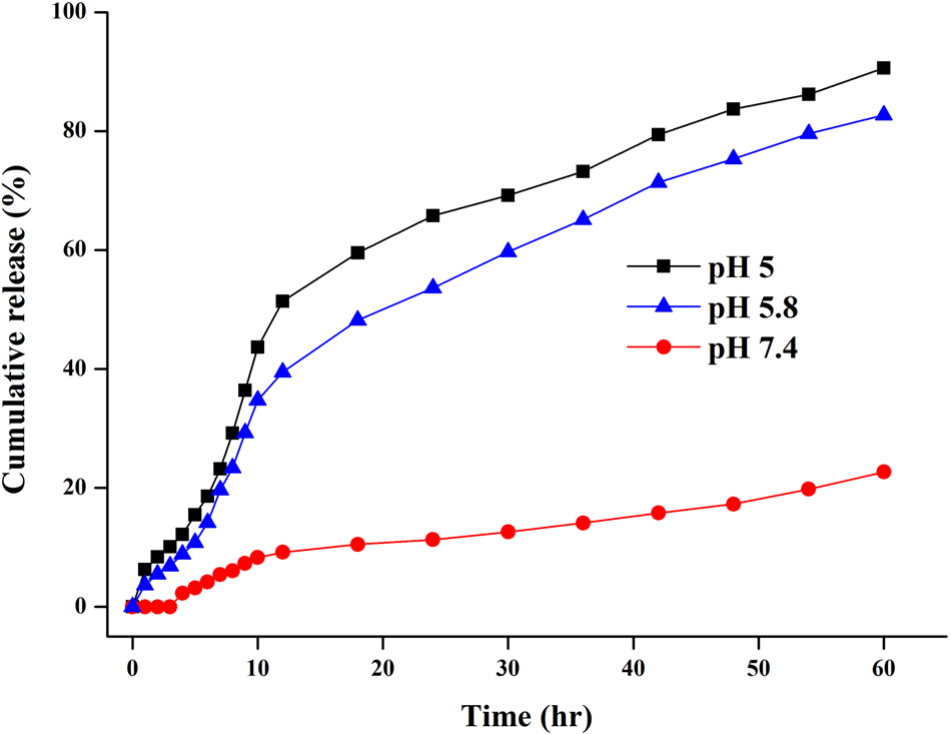
Quantitative analyses of *in vitro* release of doxorubicin at 37 °C from doxorubicin conjugated PVP-AuNPs in acetate buffer (pH 5 and pH 5.8) and 1× PBS (pH 7.4).

As shown in Fig. S3, the fluorescent intensity of Dox@PVP-AuNPs was very low due to dox was still conjugated with the surface of PVP-AuNPs. Increase the time of *in vitro* drug release conducted in dialysis membrane (12–14 kDa), the significant increase of fluorescent intensity was observed owing to release of dox from Dox@PVP-AuNPs conjugation. In contrast to time, varying the pH of solution in membrane insignificantly influences the release of dox from Dox@PVP-AuNPs. As shown in Fig. 3, lowering the pH (acetate buffer - pH 5 and PBS buffer - pH 5.8) was useful to detachment of dox but increasing the pH from 5 to 7.4 the release of dox much slower during time variation. After 60 hr incubation, the cumulative release of DOX from Dox@PVP-AuNPs at pH 5 and pH 5.8 was 91% and 83% respectively while release of DOX at pH 7.4 was only 23%.

### Cytotoxic activity

**Figure 4:**
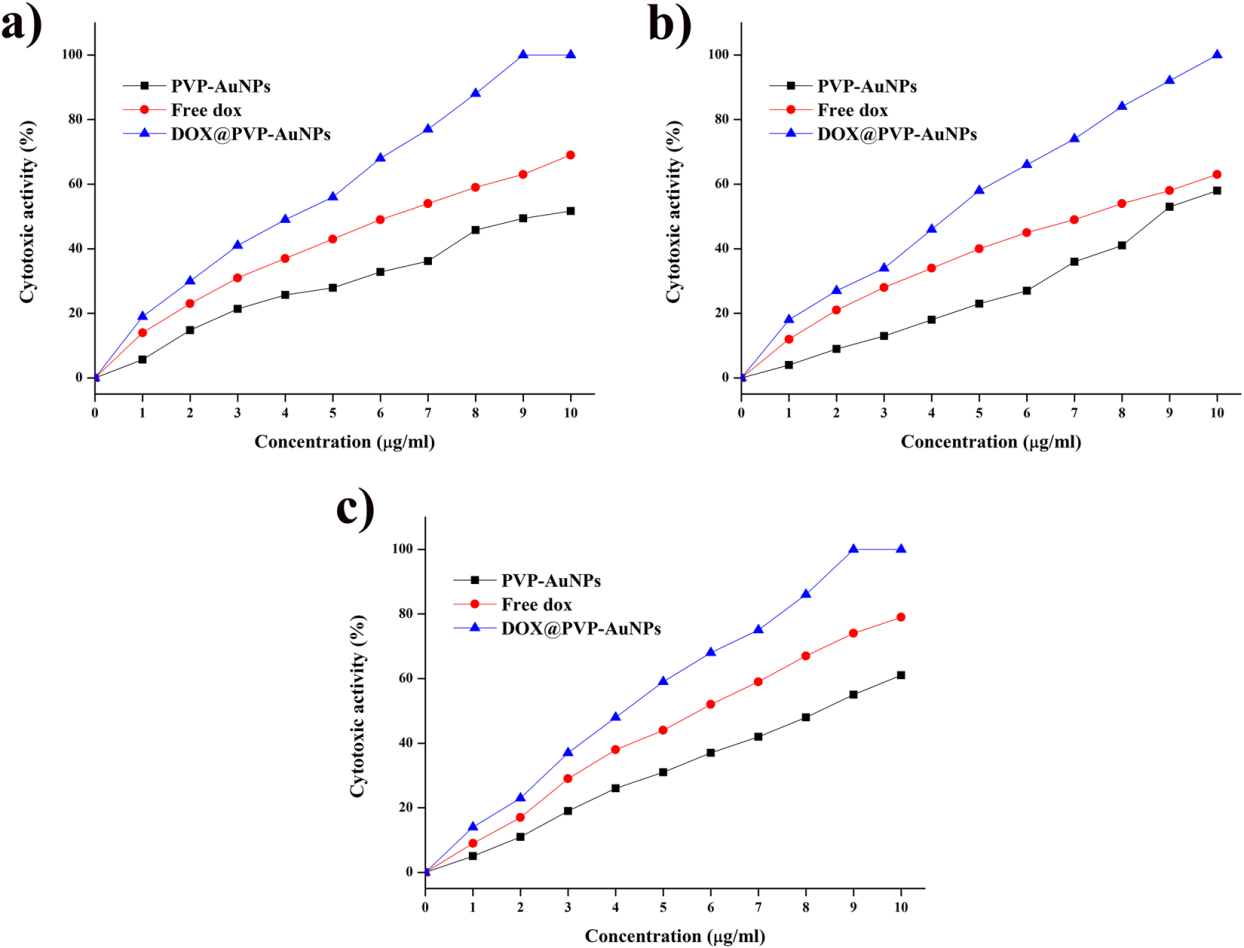
Cytotoxic activity of different concentrations (0–10 µg/ml) of PVP-AuNPs, Free Dox and Dox@PVP-AuNPs against A549 human adenocarcinoma lung cancer cells (**a**), H460 human large-cell lung carcinoma cells (**b**) and H520 human squamous cell carcinoma cells (**c**).

The analysis of cytotoxicity of a drug is one of the prominent methods for the toxicological study to find out the effect of a drug on cellular processes including apoptosis, proliferation and other metabolic responses. The cytotoxic activity of PVP-AuNPs, free Dox and Dox@PVP-AuNPs at different concentrations (0–10 µg/ml) was evaluated using an MTT assay against lung cancer cells and the results indicated that increasing the concentration increases the cell death of lung cancer cells. About 9, 10 and 9 µg/ml of Dox@PVP-AuNPs has inhibited the complete growth of A549, H460 and H540 cancer cells respectively. IC_50_ concentration for PVP-AuNPs, free Dox and Dox@PVP-AuNPs was found to be 9 µg/ml, 6 µg/ml and 4 µg/ml respectively to inhibit A549 lung cancer cells (Fig. 4a). For H460 cells, 9 µg/ml, 7 µg/ml and 5 µg/ml of the drug were found to be an IC_50_ concentration (Fig. 4b). Meanwhile, IC_50_ concentration for PVP-AuNPs, free Dox and Dox@PVP-AuNPs was found to be 8 µg/ml, 6 µg/ml and 4 µg/ml respectively to inhibit H520 cells (Fig. 4c). These IC_50_ concentration values were fixed for further study to evaluate anticancer activity of PVP-AuNPs, free Dox and Dox@PVP-AuNPs.

### Clonogenic activity of lung cancer cells

Clonogenic assay or Colony-forming assay was used to determine the long-term cytotoxic potential of PVP-AuNPs, free Dox and Dox@PVP-AuNPs (Fig. S4). A549, H460 and H520 lung cancer cells were treated with fixed IC_50_ and incubated for 10 days to calculate the colony formation. The number colonies formed in control and treated cells can be visualized from the plates by crystal violet staining, the less number of colonies were observed in PVP-AuNPs, free Dox and Dox@PVP-AuNPs treated plate compared with control plate. This finding suggested that Dox@PVP-AuNPs was effectively inhibited the proliferation of cancer cells.

### Morphological changes

Phase contrast microscopic observations of A549, H460 and H520 cells were observed after 24 hr treatment with IC_50_ concentration of PVP-AuNPs, free Dox and Dox@PVP-AuNPs. Control cells showed no significant morphological changes in human lung cancer cells. In treated cells, cells exposed to the fixed IC_50_ doses of PVP-AuNPs and free Dox showed a cell shrinkage, detachment of cells, protrusion of the membrane and reduction in size as well as cell density as compared to untreated cancer cells. The Dox@PVP-AuNPs has affected the morphology of cells such as reduces in the number of cells, damages in cell wall membrane, chromatin cleavage, compression of the cytoplasmic membrane and cell clumping was observed (Fig. S5).

### Apoptosis of cancer cells

Apoptosis of lung cancer cells induced by the appropriate concentration of PVP-AuNPs, free Dox and Dox@PVP-AuNPs was studied using AO/EB double staining assay. After 24 hr treatment, A549, H460 and H520 cells were stained with AO/EB dual staining and examined under fluorescent microscope. There is no significant cell death were observed in control samples that emitted green color fluorescence. The yellow color cells observed during the treatment of PVP-AuNPs indicated the necrotic cells. The orange and red color cells were observed while treating free Dox and Dox@PVP-AuNPs indicated that early and late apoptosis of lung cancer cells respectively (Fig. S6).

### Hochest staining

Moreover, the apoptosis was induced by Dox@PVP-AuNPs conjugates apoptosis in A549, H460 and H520 cancer cells were assessed by Hochest staining assay. The IC_50_ values of PVP-AuNPs, free Dox and Dox@PVP-AuNPs on lung cancer cells were increases the apoptosis. Apoptosis of cancer cells showed chromatin condensation, fragmentation in multiple, segregated bodies, the formation of apoptotic bodies which are visualized by bright blue fluorescence while control cells showed less blue fluorescence (Fig. S7).

### Induction of ROS generation

The ability of PVP-AuNPs, free Dox and Dox@PVP-AuNPs to induce ROS generation in cancer cells was studied using fluorescence microscopy. Cancer cells were cultured in 6 well culture plate for 24 hr and IC_50_ concentration of PVP-AuNPs, free Dox and Dox@PVP-AuNPs were exposed to cancer cells. Control and treated cells were stained with intracellular ROS indicator DCFH-DA and observed under fluorescent microscope. The micrographic images showed that treated cells increased ROS generation in drug dependent manner as compared to control (Fig. S8). After 24 hr of treatment, PVP-AuNPs, and free Dox were slightly increased the level of ROS and the maximum ROS generation was observed in 4, 5 and 4 µg/ml of Dox@PVP-AuNPs treated A549, H460 and H520 cells respectively.

### Sensitization of mitochondrial membrane potential

The effect of Dox@PVP-AuNPs conjugates and PVP-AuNPs, free Dox alone on depolarization of mitochondrial membrane was examined using Rhodamine 123 staining assay. Cells were treated for 24 hr, after treatment the cells were stained with Rhodamine 123 solution and observed under the fluorescent microscope. Treatment with Dox@PVP-AuNPs increases the cellular uptake of the fluorochrome when compared to those treated with PVP-AuNPs and free Dox (Fig. S9). These findings showed that the cells treated with Dox@PVP-AuNPs depolarize the membrane potential of mitochondria which enhances the more ROS generation in A549, H460 and H520 lung cancer cells.

### Effect on up regulation of apoptotic genes

**Figure 5:**
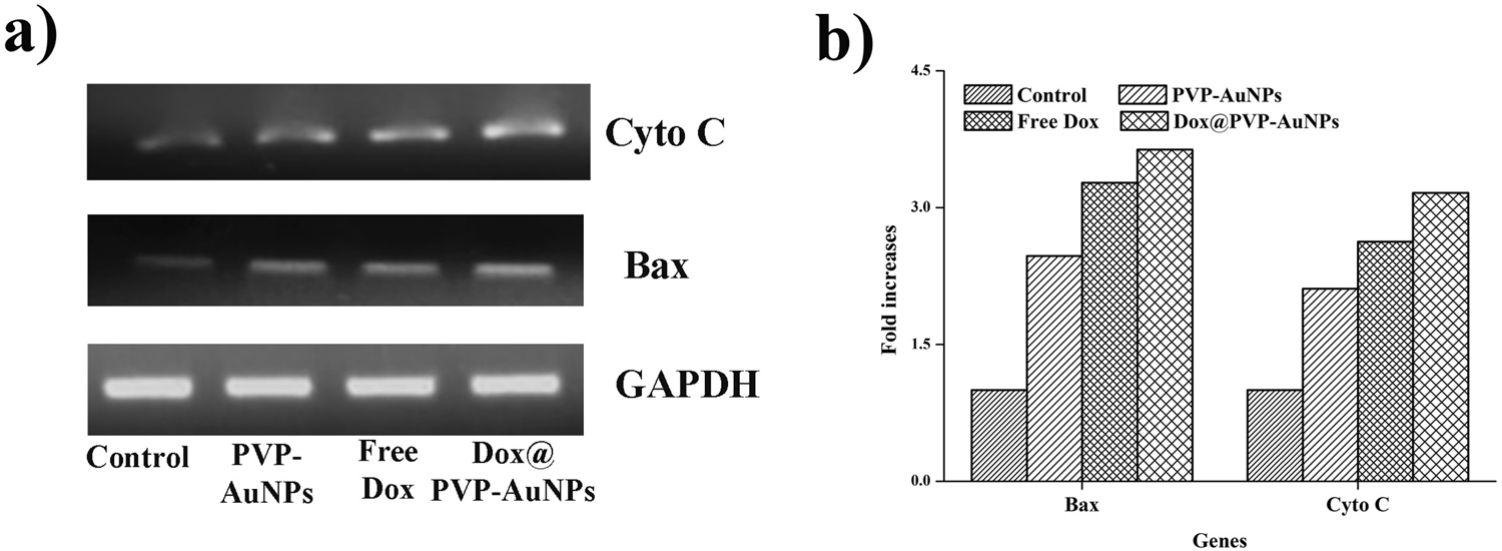
The mRNA expression of apoptotic genes such as Bax and Cyto C (**a**) was determined in A549 cells treated with IC_50_ concentrations of PVP-AuNPs, Free Dox and Dox@PVP-AuNPs. Fold increase between control and treated cells were determined using GAPDH as reference gene (**b**).

We next investigated that the expression of apoptotic genes such as Cytochrome oxidase C and Bax using RT-PCR analysis. A549 lung cancer cells were cultured for overnight and treated with IC_50_ concentration of PVP-AuNPs, free Dox and Dox@PVP-AuNPs for 24 hrs. We observed that the maximum expression of apoptotic genes such as Cytochrome oxidase C and Bax in cells treated with 4 µg/ml of Dox@PVP-AuNPs. In meantime, a significant expression of apoptotic genes was also observed in PVP-AuNPs and free Dox treated cells. There is no apparent expression was observed in untreated human lung cancer cells (Fig. 5).

### Effect on up regulation of p21 and p53

**Figure 6:**
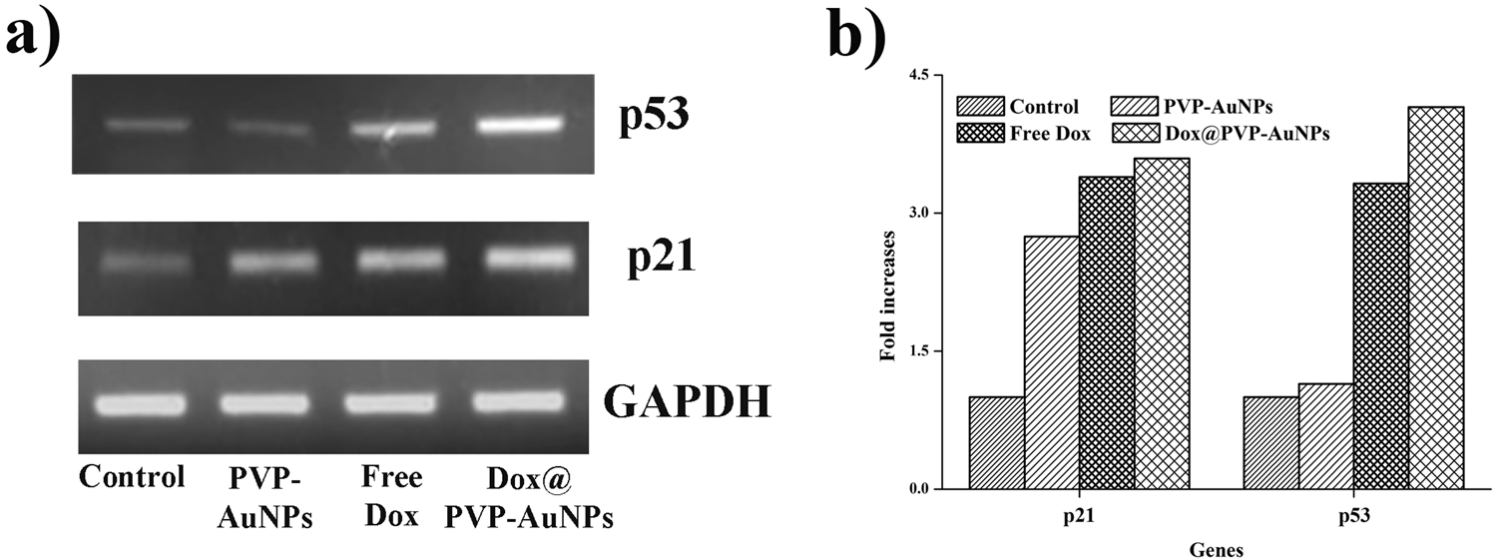
Increased expression of tumor suppressor genes p21 and p53 was observed in the treatment with IC_50_ concentrations of PVP-AuNPs, Free Dox and Dox@PVP-AuNPs against A549 human adenocarcinoma lung cancer (**a**). Quantification of gene expression based on band intensity showed as histogram using GAPDH as reference (**b**).

IC_50_ concentration of DOX conjugates as well as PVP-AuNPs and free Dox upregulates the expression of major tumor suppressor genes p21 and p53 are responsible for cell cycle arrest and p53 mediated apoptosis respectively. About 9 µg/ml concentration of PVP-AuNPs increases the expression of p21 and p53 gene while the expression level was increased in A549 cells treated with 6 µg/ml concentration of free Dox (Fig. 6). However, the maximum expression of p21 and p53 was observed in cells treated with Dox@PVP-AuNPs (4 µg/ml). From these results, it is evident that Dox@PVP-AuNPs induces intrinsic apoptotic pathway via regulating p53 and p21 which results in activation of major tumor suppressor genes.

### Cell cycle analysis

**Figure 7:**
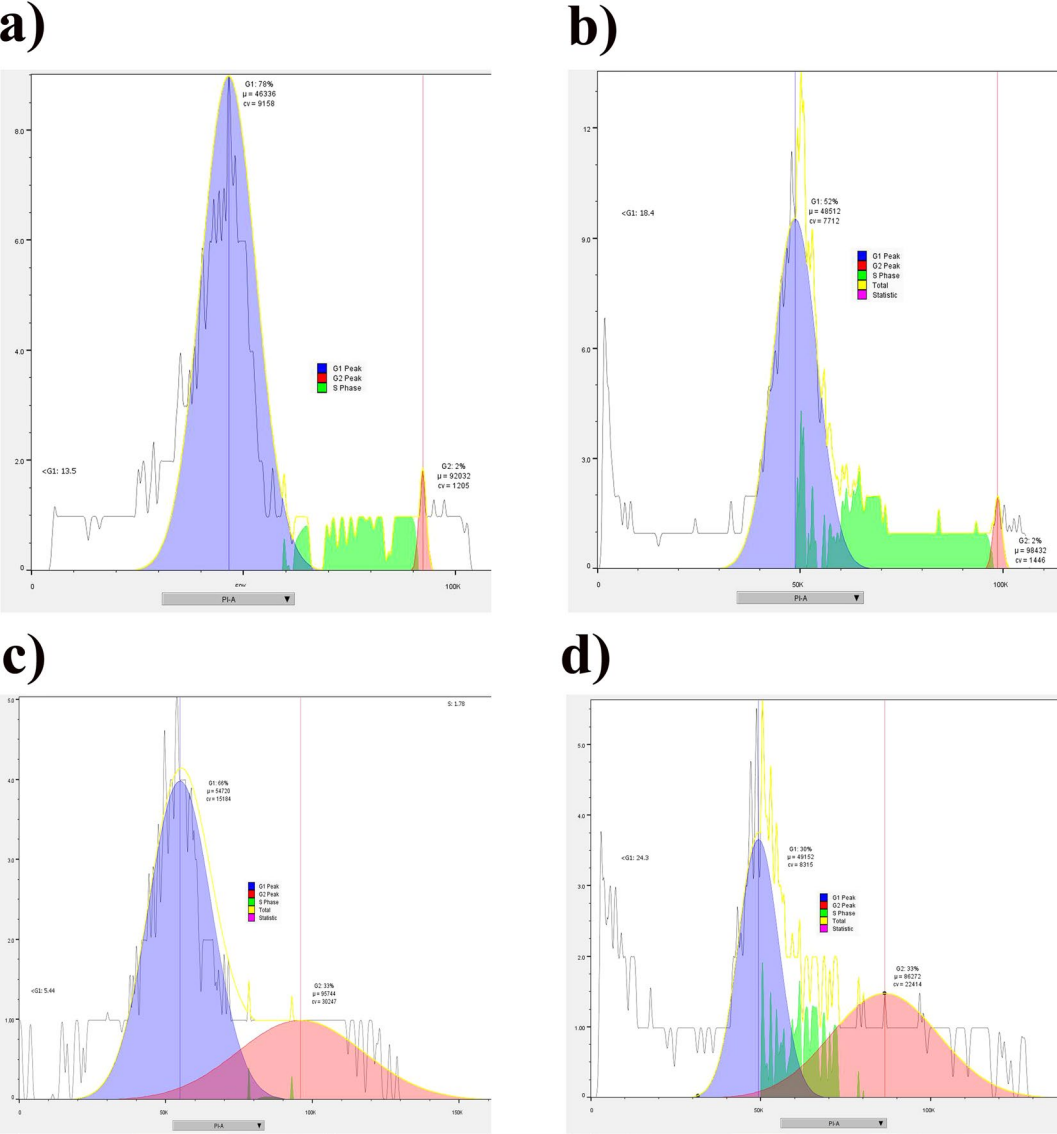
Cell cycle analysis in A549 cells treated with control (**a**), PVP-AuNPs (**b**), Free Dox (**c**) and Dox@PVP-AuNPs (**b**) by PI staining followed with flow cytometry analysis.

Further, we also investigated that whether overexpression of p21 alter the cell cycle regulation. A549 lung cancer cells were treated with PVP-AuNPs, free Dox and Dox@PVP-AuNPs and cell cycle analysis was performed using PI staining assay. Dox@PVP-AuNPs enhanced expression of p21 increase the cell cycle arrest along with the number of cells in G2 or M phase of the cell. About 9 µg/ml of PVP-AuNPs increases the cells in S phase and 6 µg/ml of free Dox in sub G0-G1 phase. Although the number of cancer cells was accumulated in G2/M cell cycle phase during the treatment of 4 µg/ml of Dox@PVP-AuNPs (Fig. 7).

### Caspase Activity

The caspases are important biochemical features of apoptotic signalling induces intrinsic apoptosis pathway. In the present study, the apoptotic response of A549, H460 and H520 cells to PVP-AuNPs, free Dox and Dox@PVP-AuNPs was examined by quantifying the activity of caspases 3 and 9. About 9 µg/ml concentration of PVP-AuNPs increases the expression of both caspases while the expression level was increased in lung cancer cells treated with 6 µg/ml concentration of free Dox (Fig. S10). However, the expression level of caspases 3 and 9 was high in the cells treated with Dox@PVP-AuNPs (4 µg/ml).Together, treatment with Dox@PVP-AuNPs induced the apoptosis in lung cancer cells through the caspase-mediated pathway.

### Effect on expression of caspase 9 and caspase 3

**Figure 8:**
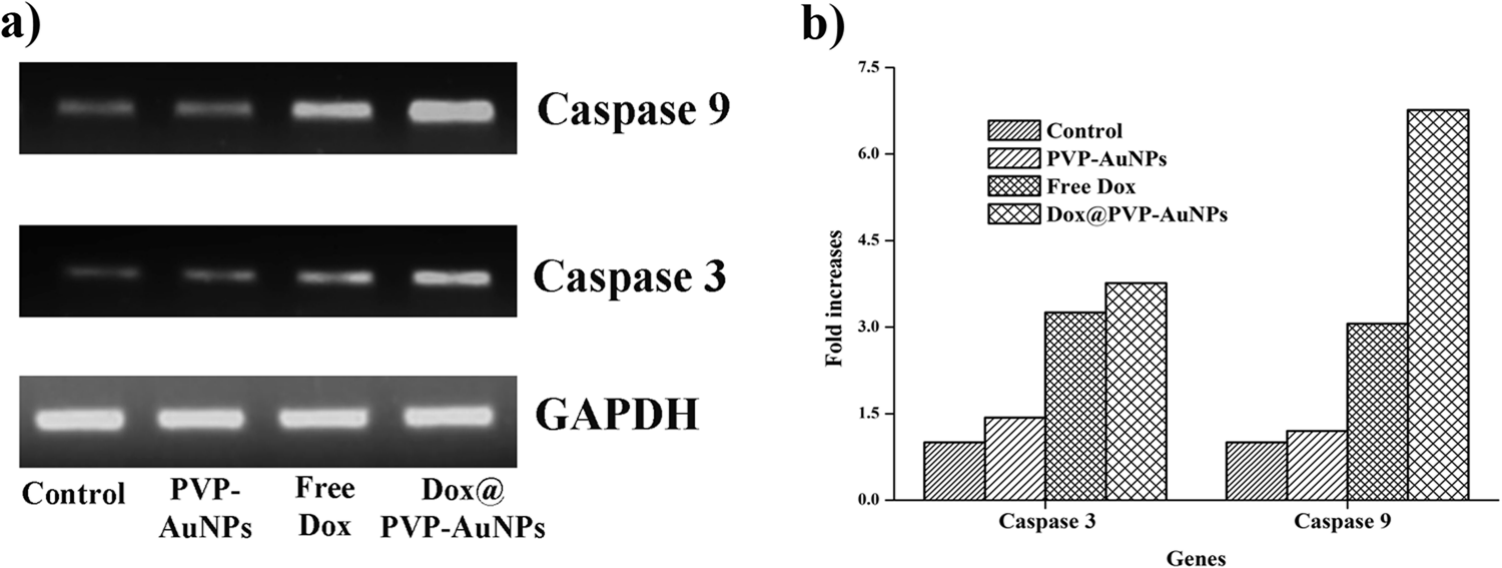
RT-PCR analysis of Caspase 9 and Caspase 3 expression in PVP-AuNPs, Free Dox and Dox@PVP-AuNPs treated A549 lung cancer cell lines (**a**). The expression level of GAPDH shown as control. Densitometric analysis was used to quantify the expression of mRNA by means of fold increases (**b**).

Finally, the expression of apoptotic genes caspase 9 and caspase 3 involving in intrinsic apoptosis pathway during the treatment of A549 lung cancer cells were examined. The results showed that increasing the concentration of Dox@PVP-AuNPs also increased the upregulation of caspase 9 and caspase 3. The 4 µg/ml concentration of Dox@PVP-AuNPs indicated an extreme level of caspase 9 and caspase 3 expressions, but it decreases while treating with PVP-AuNPs and free Dox (Fig. 8). The outcome of these findings suggested that the Dox@PVP-AuNPs significantly involve in the upregulation of caspase 9 and caspase 3 that leads to dysregulation of the death receptor and mitochondrial signalling pathway.

## Discussion

Lung cancer is the most common cause of cancer-related death worldwide. Among NSCLC accounts for >85% of primary lung cancers, and approximately two-thirds of NSCLC patients are diagnosed at an advanced stage^16^. Since the chemotherapy and radiation therapy has more side effects, it’s an urgent need for treatment of lung adenocarcinoma. In medicine, nanotechnology has glowed a promptly increasing interest as it promises to resolve a number of issues associated with chemoprevention^17^. Among drug-loaded polymer nanoparticles are promising drug delivery systems for the treatment of severe diseases such as cancer, infections, and neurodegenerative disorders^18^. In the present study, we also demonstrated the anticancer activity doxorubicin conjugated with PVP coated AuNPs against A549, H460 and H520 lung cancer cells and results indicated that Dox@PVP-AuNPs effectively inhibit the proliferation of lung cancer cells and induces p53 mediated mitochondria dependent apoptosis.

Initially, the synthesis of PVP stabilized AuNPs was confirmed by visual observation with the appearance of color change from yellow to dark violet in the reaction mixture contains HAuCl_4_ and NaBH_4_. AuNPs are usually produced by the addition of reducing agent to a solution of gold chloride causes reduction of gold ions and accumulation of Au atoms into AuNPs^19^. According to Brust–Schiffrin procedure, NaBH_4_ was found to be the most important reductants and used to condense transition metal salts to metal NPs in the presence of CTAB^20^. Further synthesis of AuNPs was confirmed by absorption peak at 525 nm is due to SPR peak and dependent on particle size^21^. By the addition of PVP with AuNPs increases the stability of AuNPs is due to the high surface active properties of PVP and solubility in water as well as pharmaceutically acceptable solvents, hence PVP is considered to be the model polymer as a metal nanoparticle stabilizer for the purpose of drug delivery systems^22^.

Further, the size of PVP-AuNPs was determined and results indicated that the addition of PVP with AuNPs reduces its size as well as increases its stability. Miao *et al*.^23^ reported that PVP could increase excellent stability and dispersion of AuNPs in aqueous solution. Owing to the coating of AuNPs with PVP molecules, PVP-AuNPs are mostly in faceted particles with diameters of 5 to 20 nm in size^24^. The polydispersity nature of nanoparticle size is due to the presence of size and shape regulating agent PVP in colloidal solution^25,26^. The monomer and amide group in PVP can act as a capping agent for AuNPs by electrostatic interaction and intermolecular bonding between hydroxyl group (AuNPs) and cyano group (PVP)^27^. The presence of monomer and amide groups in PVP can be adsorbed by AuNPs on the surface and impede AuNPs to get close contact with the polymer which enhances the stability through steric stabilization mechanisms^28^.

Further DOX was conjugated with PVP-AuNPs by the formation of hydrazone bonds between hydrazine moiety of PVP-AuNPs and ketonic groups of DOX^29^. The formation of Dox conjugated with PVP-AuNPs was confirmed by fluorescence quenching studies and results showed that the characteristic bands of free Dox molecule vanished after conjugated with PVP-AuNPs. Due to the effect of nanosurface energy transfer (NSET) between Dox and PVP-AuNPs, the fluorescence intensity of Dox was quenched. As reported earlier^30^, an absorption band concerning to SPR of PVP-AuNPs appeared at 525 nm on completion of conjugation between Dox and PVP-AuNPs. Further, the degradation of hydrazone bonds of Dox@PVP-AuNPs will result in the release of doxorubicin from the nanoparticles was demonstrated at different pH conditions. Ruan *et al*.^31^ reporting that lowering the pH from 7.4 to 5 leads to release of Dox from Dox-AuNPs complex and increases the fluorescence intensity, suggesting low pH was useful for elevating the hydrolysis of hydrazone that led to the detachment of DOX.

The cytotoxic activity of PVP-AuNPs, free Dox and Dox@PVP-AuNPs against human lung cancer was evaluated using MTT assay. The significant difference in the viability of cancer cells when cultured in the presence of PVP-AuNPs, free Dox and Dox@PVP-AuNPs. Among Dox@PVP-AuNPs showed excellent cytotoxic activity than PVP-AuNPs and free Dox that clearly confirms the conjugation of drug and AuNPs has the significant effect on inhibition of viability of cells. The cytotoxicity of doxorubicin conjugated with polymer coated AuNPs to breast cancer cells was higher than free Dox indicating that polymer-conjugated micelles have the ability to selectively target the cancer cells^32^. The pH responsive Dox@PVP-AuNPs enhanced drug release in cancer cells and rapidly released the tethered drug into the cells. As a result, delivery of doxorubicin with Dox@PVP-AuNPs can rapidly increase the concentration of free drug in A549 cells, thereby improving its cytotoxicity^33^.

Elevated rates of ROS promotes many aspects of tumor development and progression, but tumor cells also express increased levels of antioxidant proteins to detoxify from ROS^34^. Disproportional increase in intracellular ROS by Dox@PVP-AuNPs complex can induce cancer cell cycle arrest, senescence and apoptosis. Moreover, this continuous ROS generation can affect severe cellular damage to mitochondrial membrane and increases intercellular oxidative stress in mitochondria which could be evidence for inducing intrinsic apoptosis based on the release of cytochrome C into cytosol^35^. Moreover increased ROS causes DNA damage by precisely oxidizing its bases and sugar backbone that leads to triggering the DNA damage response (DDR) checkpoint and arrest proliferation^36^. Such DDR causes the endurance of signals and leads to cell cycle arrest, apoptosis or cell senescence^37^.

In response to genotoxic stress, DDR signalling pathway is activated, causing cell cycle arrest to allow the correction of the damage and to maintain genomic integrity^38^. The DDR is a cellular defence mechanism that integrates genotoxic event detection to the activation of checkpoint pathways to arrest cells in different phases of the cell cycle to facilitate DNA repair or induce apoptosis and eliminate damaged cells^39^. In the present study, Dox@PVP-AuNPs complex arrests the cell cycle of human lung cancer at G2/M phase after 24 hr treatment. The product of the p53 gene plays an important role in DDR, where it works as a tumor suppressor mainly involved in the transcriptional regulation of a large number of growth arrest and apoptosis related genes including p21^40,41^. The tumor suppressor p53 functions predominantly act as a transcription factor by activating and downregulating gene expression, leading to cell cycle arrest and apoptosis^42^. To correlate with the previous report, Dox@PVP-AuNPs upregulates the expression of p53 and p21 that initiates cell cycle arrest and caspase mediated apoptosis. p53 transcriptionally binds with promotor region of p21 to establish a G1 phase of cell cycle without activation of cell death pathways^43^.

In other hands, the inappropriate production of ROS in mitochondria is linked to a mitochondrial membrane depolarization that causes cytochrome C release, an irreversible event that leads to the activation of caspases and cell death^44^. ROS generated by Dox@PVP-AuNPs in mitochondria upregulates the expression of apoptotic genes such as cytochrome c and Bax and the results lead to induce the mitochondria mediated apoptosis in lung cancer cells. Numerous studies stated that the release of cytochrome C from mitochondria that promotes massive activation of executioner caspases-3 is the critical checkpoint in cell commitment to death^45–47^. In the present study Dox@PVP-AuNPs complex enhances the expression of caspase 9 and caspase 3 and AO/EB staining assay confirmed the apoptosis in lung cancer by emitting red color fluorescence in Dox@PVP-AuNPs chromatin condensation and formation of apoptotic bodies by bright blue fluorescence. Further, the caspase enzymatic assay also confirms the Dox@PVP-AuNPs complex enhances the apoptosis in lung cancer cells through caspase-mediated pathway.

In conclusion, AuNPs was synthesized using CTAB method and surface properties were improved with PVP which were characterized using spectroscopic and microscopic techniques. These PVP-AuNPs were used as a successful carrier for delivery of doxorubicin for treatment of lung cancer. The pH responsive of drug release from Dox@PVP-AuNPs has potential cytotoxic activity, ROS generation, sensitization of mitochondrial membrane that leads to induce intrinsic apoptosis in lung cancer cells. Additionally, Dox@PVP-AuNPs upregulates the expression of numerous tumor suppressor genes showed the capability of the potential anticancer agent in cancer treatment (Fig. S12). These findings could support the development of drug delivery system targeting intrinsic apoptosis pathway.

## Materials and Methods

### Chemicals and reagents used

Dulbecco’s modified Eagle’s medium (DMEM), fetal bovine serum (FBS), penicillin/streptomycin, DMSO (cell culture grade), MTT (dimethylthiazolyltetrazolium bromide), Acridine orange, Ethidium bromide, Chloroauric acid (HAuCl_4_), 1× Phosphate buffer saline (PBS) and Glutamine were purchased from HiMedia Laboratories, Mumbai, India. Cetyl trimethylammonium bromide (CTAB), 2′,7′ -Dichlorofluorescein-diacetate (DCFH-DA) and Rhodamine-123 dye were purchased from Sigma-Aldrich, USA. Propidium Iodide was purchased from Thermo Fisher Scientific, India.

### Synthesis of AuNPs

AuNPs stabilized with CTAB was synthesized by the previously reported method as described by Tinguely *et al*.^48^ a standard reduction of HAuCl_4_ in NaBH_4_ as a reducing and capping agent. Solutions of 100 ml HAuCl_4_ (1 × 10^−3^ M), 100 ml of CTAB (0.08 M) and 100 ml of NaBH_4_ (2 × 10^−3^ M) was prepared and incubated for 30 min at 4 °C in dark place. The typical reaction containing 34 ml of HAuCl_4_ and 10 ml of CTAB was prepared in Milli-Q water and kept in magnetic stirrer (70 °C) for 10 min. A specific amount of NaBH_4_ (6 ml) was added dropwise into precursor solution under dynamic stirring and kept in the ice bath for 30 min. After that, solution mixture was heated to 60 °C to activate the reduction of HAuCl_4_. For all reactions, the reaction containers were covered with aluminium foil and reduction of HAuCl_4_ by NaBH_4_ was examined by the change in color. The synthesized AuNPs were confirmed using UV spectrophotometer and crystalline nature, size and stability of CTAB mediated prepared AuNPs was further characterized.

### Preparation of PVP-stabilized AuNPs (PVP-AuNPs)

To prepare hydrophobically modified PVP-AuNPs, AuNPs were conjugated with PVP by following previous method^49^. Briefly, 2 ml of AuNPs (1 mg/ml) was dissolved in Milli-Q water and centrifuged at 12000 rpm for 10 min. the supernatant was discarded. AuNPs was resuspended in Milli-Q water (2 ml) and 2 ml of PVP (1 mg/ml) was added dropwise under dynamic stirring at magnetic stirrer at 60 °C. After 3 hr of stirring, the free PVP was removed by ultracentrifugation (16000 rpm, 15 min.) and dissolved into 2 ml of milli-Q water at the final concentration of 1 mg/ml concentration.

### Characterization

The crystalline nature of PVP-AuNPs was examined using high resolution transmission electron microscopy (HRTEM) equipped with selected area electron diffraction (SAED). The structure and phase purity of synthesized PVP-AuNPs were analysed by powder X-ray diffraction. The size and stability of PVP-AuNPs were determined using dynamic light scattering (DLS) and zeta potential measurement respectively.

### Nuclear Magnetic Resonance (NMR)

NMR spectroscopy was performed using Bruker DPX-300 and Bruker DRX-500 instruments, with dimethyl sulfoxide (DMSO)-d6 as the solvent. To prepare samples for analysis, 0.005 g (for ^1^H NMR) or 0.039 g (for ^13^C NMR) of PVP-AuNPs was dissolved in 0.6 mL DMSO-d6 and added to a clean dry NMR tube, which was then capped and protected from light. NMR integral errors are ±10%.

### Conjugation of doxorubicin with PVP-AuNPs

A simple dialysis method was used to conjugate doxorubicin with PVP-AuNPs (Dox@PVP-AuNPs). Typically, 1 mg of prepared PVP stabilized AuNPs was dissolved in 1 ml of milli-Q water and mixed with 0.2 mg of DOX containing water (pre-treated with 25 µl of 0.15 mM triethylamine). The mixture was sonicated five times for 2 min. and followed by dialysis at overnight. After filter with 0.8 µm syringe filter to remove unconjugated DOX, the mixture was lyophilized and dispersed in milli-Q water at the final concentration of 1 mg/ml^30^.

### Kinetics of DOX release from Dox@PVP-AuNPs conjugates

Drug release studies of PVP-AuNPs formulations of DOX were conducted using previously described technique^50^. To measure the release of DOX profile, NP solutions at a concentration of 100 µg/ml (2 ml) were split into dialysis membrane (HiMedia) with molecular cut-off of 10 kDa and subjected to dialysis against 25 ml acetate buffer (pH 5 and pH 5.8) and 1× phosphate buffer saline (pH 7.4) with gentle stirring at 37 °C. Buffer solutions were changed periodically during the process. At the indicated times, 5 ml of solution was removed from the dialysis membrane and equal volume of fresh buffer solutions (appropriate pH) was replaced. The collected solutions were analysed to quantify the amount of DOX released using UV-Spectrophotometer at 490 nm.

### Cancer cell line

Human lung adenocarcinoma cells (A549), human large-cell lung carcinoma cells (H460) and human squamous cell carcinoma cells (H520) were purchased from National Centre for Cell Science (NCCS), Pune, India. The cells were cultured in Dulbecco’s Modified Eagle Medium (DMEM) supplemented with fetal bovine serum (10%), 1% antibiotic (50,000 units/l of penicillin and 50 mg/l of streptomycin) and 5 mM glutamine. Cultures were incubated in tissue culture flasks at 37 °C in 5% CO2 and 95% relative humidity. Culture media was replaced at least twice a week.

### Cytotoxic activity

The cytotoxic activity of Dox@PVP-AuNPs against human lung cancer cell lines was determined by following previously described method^51^. Lung cancer cells were seeded in a 96-well plate (10 × 10^4^ cells per well) and incubated for 24 hr. The cells were treated with different concentration of PVP-AuNPs, free Dox and Dox@PVP-AuNPs and incubated for 24 hr with the supply of 5% CO_2_ and 95% relative humidity. The cells without drugs were treated with DMSO and used as a control. After 24 hr of incubation, 25 µl of an MTT solution (5 mg/ml) was added to wells and plate was further incubated for 4 hr. The culture medium in each well was replaced with 100 µl of DMSO and plate was gently agitated for 15 min. Absorbance was measured at 570 nm using an enzyme-linked immunosorbent assay plate reader. On comparing with untreated cells, the percentage of viable cells in treated culture was calculated and IC_50_ was taken for further study.

### Clonogenic assay

The clonogenicity or colony formation of cancer cells was evaluated using crystal violet staining method by previous method^52^. The cells were harvested and seeded in six well culture plates at a density of 250 cells/well containing 2 ml of culture medium. Cells were kept for the adherent at the surface of culture plate for 16 hr. After incubation, cells were treated with PVP-AuNPs, free Dox and Dox@PVP-AuNPs and incubated for 7 days at 37 °C with 5% CO2. After 7 days, the cells were washed using 1× PBS, fixed with ice cold methanol and stained with crystal violet for 5 min at 26 °C. The plate was washed gently with distilled water and air dried. The plate was photographed and the effectiveness of plating was calculated for control as well as treated cells.

### Morphological changes

For morphological change analysis, A549, H460 and H520 cells were cultured in 6-well plates. The cells were treated with IC_50_ concentration of PVP-AuNPs, free Dox and Dox@PVP-AuNPs for 24 hr. After treatment, the cells were washed twice with PBS and the cells were fixed with ethanol:acetic acid solution (3:1). Finally, the plates were observed under bright field inverted light microscopy (Nikon, Japan) at 40× magnification. The morphological changes of lung cancer cells were documented^53^.

### AO/EB staining of apoptotic cells

Cells were seeded on glass coverslips in 6 well culture plates at 1 × 10^5^ cells per well density and grown 24 hr at 37 °C in 5% CO_2_ incubator. IC_50_ concentration of PVP-AuNPs, free Dox and Dox@PVP-AuNPs and vehicle control (DMSO) for 24 hr. After trypsinization, each cell was washed two times with 1× PBS and the plate was further incubated for 5 mins with AO/EB (50 µg/ml in 1× PBS). Cells were immediately visualized by fluorescent microscopy (Nikon 80i, Japan) at 40× magnification with excitation filter 480/30 nm. Acridine orange is taken up by both live and dead cells and emits green fluorescence, whereas ethidium bromide stains were observed only by dead cells which lost their membrane integrity and emits red fluorescence^54^.

### Hoechst staining

The effect of apoptotic on A549, H460 and H520 cells was performed using Hoechst 33258 staining method. The cells were seeded in 6-well tissue culture plates (5 × 10^4^ cell/ml). After incubations overnight, the medium was removed and replaced with fresh medium plus 10% FBS and then treated with IC_50_ concentration of PVP-AuNPs, free Dox and Dox@PVP-AuNPs for 24 hr. The culture medium was removed and the cells were fixed in 4% paraformaldehyde for 10 min. The cells were washed twice with PBS and were consequently stained with 0.5 mL of Hoechst 33258 staining for 5 min. The stained nuclei were washed twice with PBS and observed under the fluorescent microscope at 350 nm excitation and 460 nm emissions^55^.

### Measurement of ROS generation

The generation of reactive oxygen species (ROS) in cells was measured using a ROS sensitive fluorescent dye, 2,7-dichlorodihydrofluorescein diacetate (DCFH-DA) by previously reported method^52^. This dye can be oxidized to 2′,7′-dichlorofluorescein (DCF) by ROS and exhibits increased green fluorescence intensity. Briefly, the cultured lung cancer cells were treated with PVP-AuNPs, free Dox and Dox@PVP-AuNPs for treatment of 24 hr. After treatment, the cells were washed twice with 1× PBS and fixed in 4% paraformaldehyde for 10 min. Then cells were stained with 10 mM concentration of DCFH-DA for 5 min and the levels of intracellular ROS were examined under a fluorescence microscope at 485 nm excitation and 520 nm emission.

### Assessment of mitochondrial membrane potential (Δψm)

The Δψm level was measured using rhodamine 123, a mitochondrial-specific fluorescent dye according to a previous report with modifications^56^. Briefly, cells (5 × 10^5^ /well) were seeded in 6 well optical plates (Greiner Bio-One, Dusseldorf, Germany). After 24 hr, the old medium was replaced with fresh medium containing PVP-AuNPs, free Dox and Dox@PVP-AuNPs and incubated for further 24 hr at 37 °C. After treatment, the cells were washed three times with 1× PBS and incubated with 1 ml of Rhodamine 123 solution (10 mg/ml in 1× PBS) for 30 min at room temperature. Fluorescence intensity was measured using fluorescent microscope at excitation and emission wavelengths of (Ex/Em) 530 nm/580 nm.

### Cell cycle analysis

A549 cells were seeded in 6-well plates for 24 hr at 1 × 10^6^ cells of density per well. After 24 hr, cells were exposed to DMSO (vehicle control), PVP-AuNPs, free Dox and Dox@PVP-AuNPs for 24 hr. Then the cells were harvested and washed with ice-cold 1× PBS buffer, fixed with 70% alcohol at 4 °C for overnight. For cell cycle analysis, the cells were stained with 50 μg/ml propidium iodide (PI) in the presence of 1% RNAase A (10 μg/ml). After 1 hr incubation at 37 °C, the cells were analyzed by a flow cytometry using Beckman Coulter EPICS XL-MCL (Brea, USA). The percentage of cells in each phase of cell cycle was determined using FlowJo software (Ashland, US).

### Caspase enzymatic activity assay

A quantitative enzymatic activity assay was carried out according to the instructions of the manufacturer for the colorimetric assay kit (Abcam, Germany). After treatment with DMSO (vehicle control), PVP-AuNPs, free Dox and Dox@PVP-AuNPs for 24 hr, the cells were washed with PBS and centrifuged at 1500 rpm for 5 min to remove the media The cell pellets were lysed with 50 μl cold lysis buffer and incubated on ice for 1 h. The protein concentrations in each tube were quantified using the Bradford method. 50 μl of 2× reaction buffer was added to 50 μl supernatant containing 200 μg protein in each tube. Subsequently, 5 μl caspase substrate was added and incubated at 37 °C for 4 h in dark. Finally, the samples were read at 405 nm in a microplate reader (Universal Microplate reader, Biotech, Inc., USA).

### Gene expression analysis - Reverse Transcriptase PCR

A549 human adenocarcinoma lung cancer cells were treated with IC_50_ concentration of PVP-AuNPs, free Dox, Dox@PVP-AuNPs and DMSO (vehicle control). Total RNA was isolated using TRIzol reagent (Invitrogen, USA) as recommended by the manufacturer. After isolated, the amount of RNA was quantified and an equal amount of total RNA was reverse transcribed to cDNA using random hexamer primer as followed by manufacturer protocol (First Strand cDNA synthesis kit, Thermo Scientific, USA). Equilibrated amounts of cDNA was amplified using GENEITM PCR master mix (Genei-Merck, India) and specific primers for the genes were used (Table S1). Amplification was carried out with first denaturation step at 94 °C for 2 min, 35 cycles of denaturation at 94 °C for 30 sec, annealing at appropriate Tm specific for the primers for 1 min^16^. Relative expression levels of the target genes were normalized with the control gene GAPDH.

## Electronic supplementary material

Supplementary information
