# Supplementary material for: Target delivery of doxorubicin tethered with PVP stabilized gold nanoparticles for effective treatment of lung cancer
Source: Sci Rep. 2018 Feb 28;8:3815. doi: 10.1038/s41598-018-22172-5 (PMC5830607; doi:10.1038/s41598-018-22172-5)
Supplement: Supplementary file 1 — Supplementary information [file 41598_2018_22172_MOESM1_ESM.doc]

**Supplementary Information**

**Target delivery of doxorubicin tethered with PVP stabilized gold nanoparticles for effective treatment of lung cancer**

**Vaikundamoorthy Ramalingam^1^, Krishnamoorthy Varunkumar^2^,**

**Vilwanathan Ravikumar^2^ and Rajendran Rajaram^1^**

**Table S1 List of forward and reverse primers used to study the expression profile of apoptotic genes and its amplicon size**

| **Apoptotic genes** | **Forward Primer** | **Reverse Primer** | **Amplicon size (bp)** | **Annealing temp (°C)** |
| --- | --- | --- | --- | --- |
| **GAPDH** | 5ˈ-CTCATGACCACAGTCCATGCCATC-3ˈ | 5ˈ-CTGCTTCACCACCTTCTTGATGTC -3ˈ | 272 | 56 |
| **Bax** | 5ˈ-ACCAAGAAGCTGAGCGAGTGT-3ˈ | 5ˈ-ACAAACATGGTCACGGTCTGC-3ˈ | 332 | 57.5 |
| **Cyt C** | 5ˈ-TTTGGATCCAATGGGTGATGTTGAG-3ˈ | 5ˈ-TTTGAATTCCTCATTAGTAGCTTTTTTGAG-3ˈ | 364 | 59.7 |
| **p53** | 5ˈ- TGACACGCTTCCCTGGATTG -3ˈ | 5ˈ- GCTGCCCTGGTAGGTTTTCT -3ˈ | 367 | 62 |
| **p21** | 5 ˈ- ACCGAGGCACTCAGAGGAG-3ˈ | 5ˈ- ATCTGTCATGCTGGTCTGCC-3ˈ | 471 | 60 |
| **Caspase 9** | 5ˈ-CTTTCTGGGCACGTGAGGTT-3ˈ | 5ˈ-CCTCCGCCAGAAAGGTACAG-3ˈ | 482 | 60 |
| **Caspase 3** | 5ˈ-CTGTGGCTGTGTATCCGTGG -3ˈ | 5ˈ-CTGAGGTTTGCTGCATCGAC -3ˈ | 379 | 60 |

**Fig. S1** High resolution transmission electron microscopic (HRTEM) image of AuNPs scale - 5 nm (a) and scale - 1 nm (b), distribution of AuNPs (c) and SAED pattern of AuNPs (d).

| a)  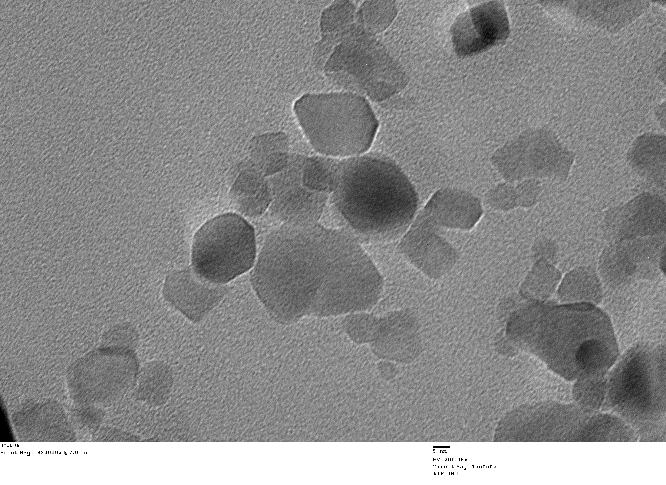 | b)  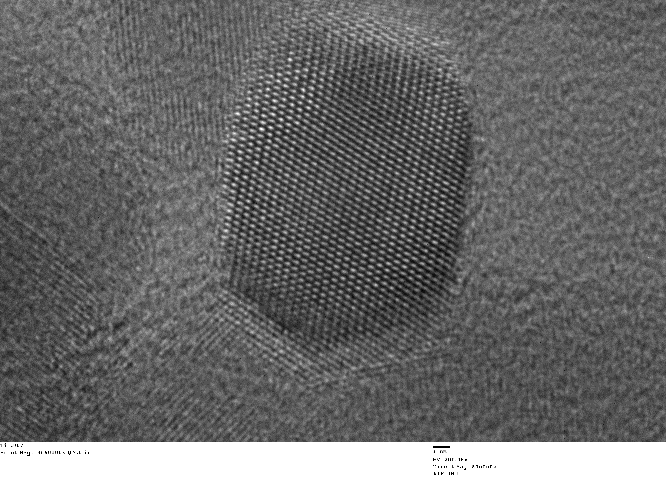 |
| --- | --- |
| c)  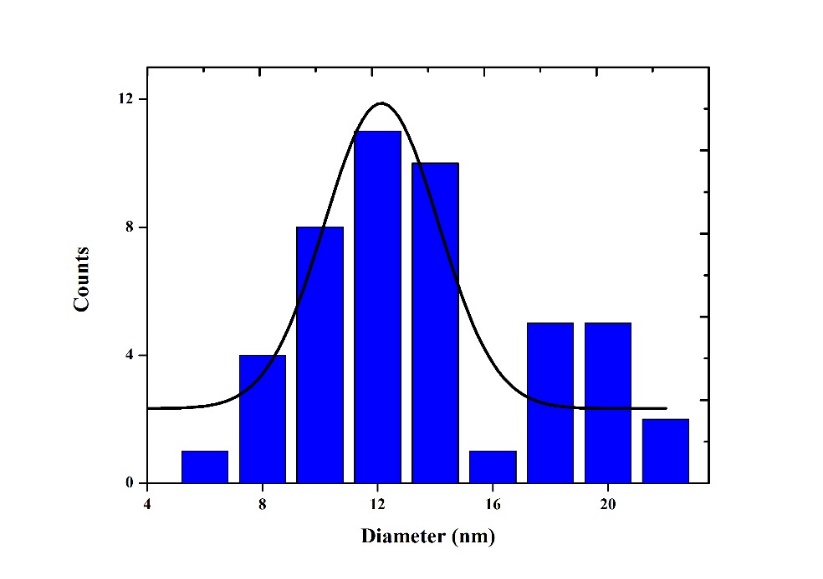 | d)  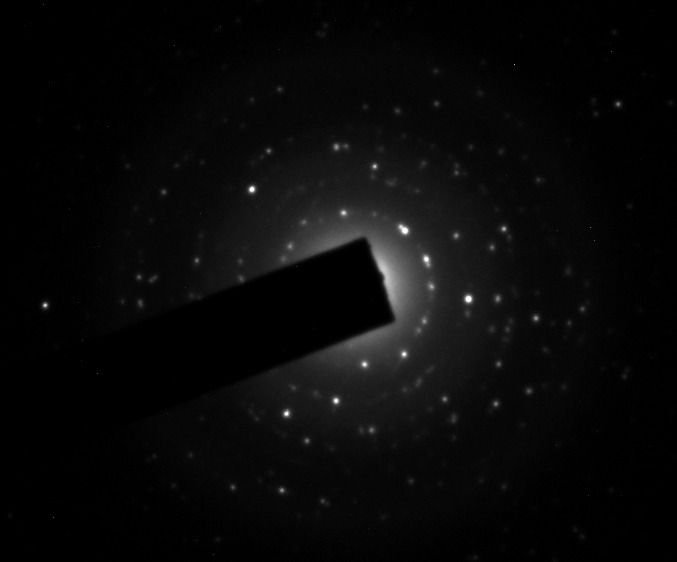 |

**Fig. S2** ^1^H NMR (a) and ^13^C NMR (b) spectrum of PVP-AuNPs

| a)  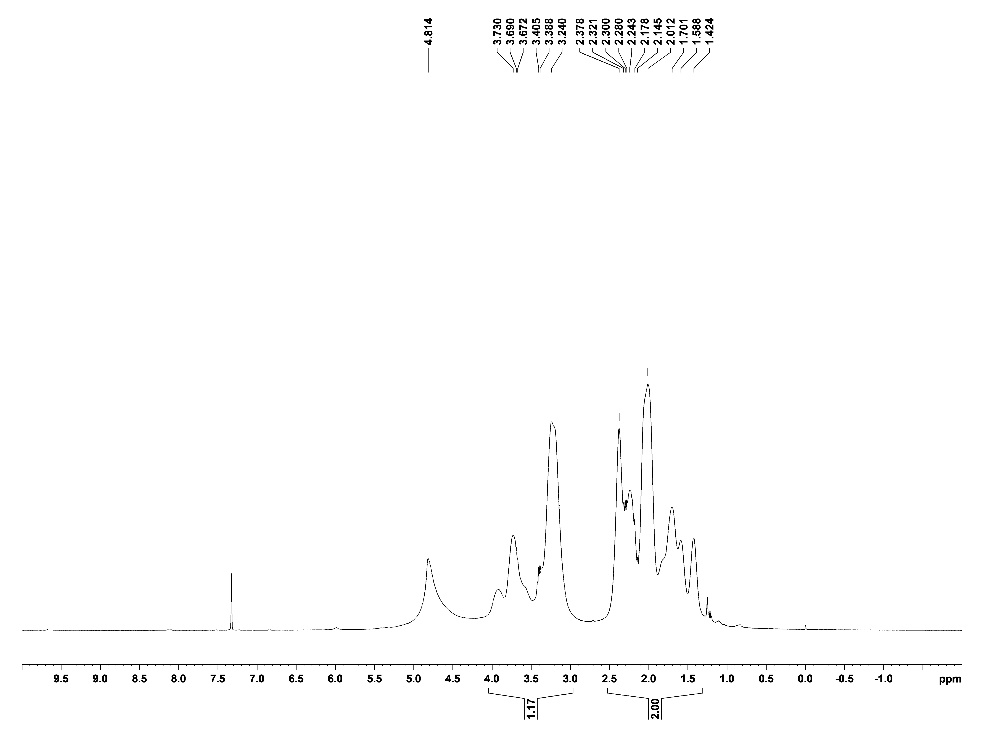 |
| --- |
| b)  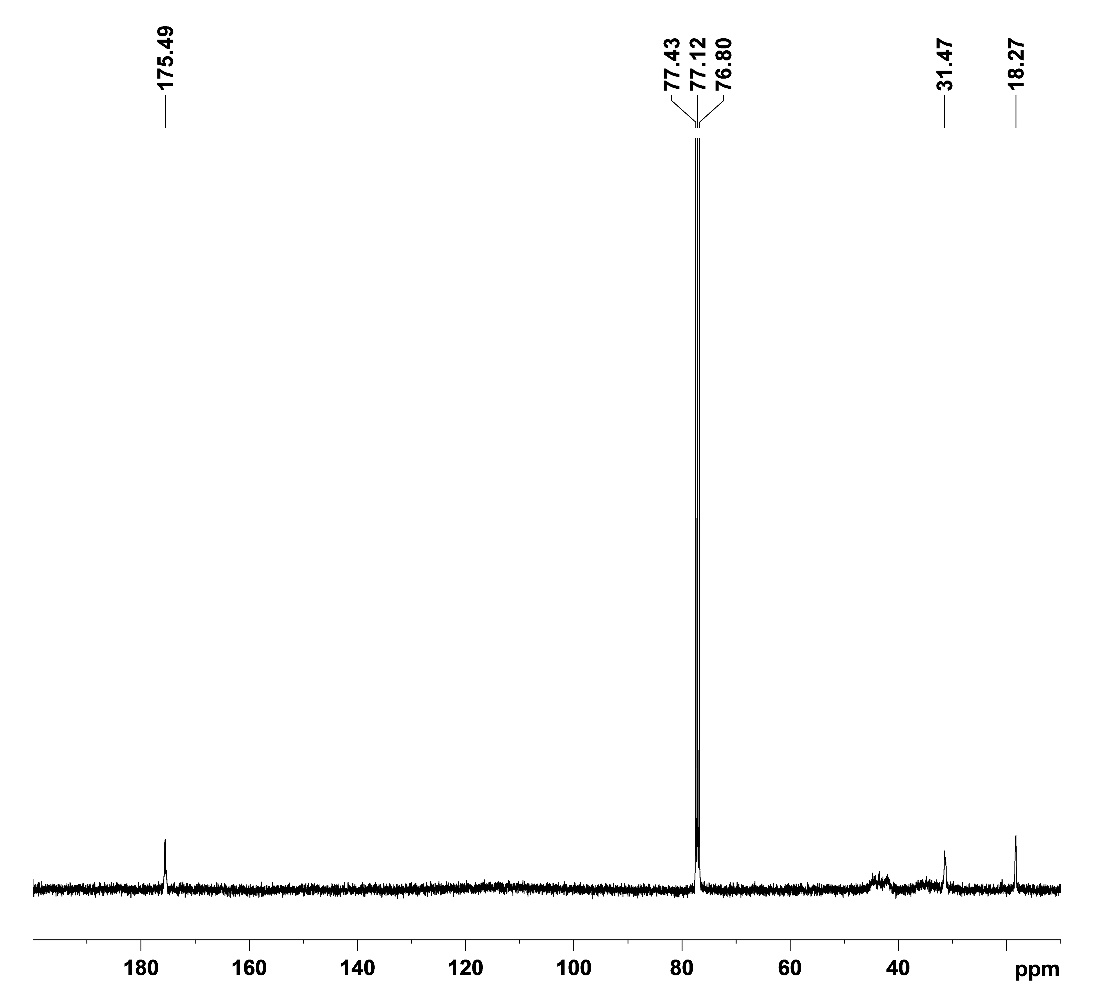 |

**Fig. S3** Fluorescence studies of Dox@PVP-AuNPs conjugates. (a) Fluorescence intensity of free dox and Dox@PVP-AuNPs. (b) Fluorescence emission spectra of Dox (5mg/ml) in aqueous solution recorded after reaction with PVP-AuNPs at different concentrations from 0 to 100 µg/ml. Emission wavelength = 595 nm. (c) UV Vis excitation spectra of free dox and PVP-AuNPs (d) UV Vis excitation spectra of PVP-AuNPs (5 µg/ml) after reaction with dox at different concentration from 0 to 10 µg/ml. Excitation wavelength = 465 nm.

| a)  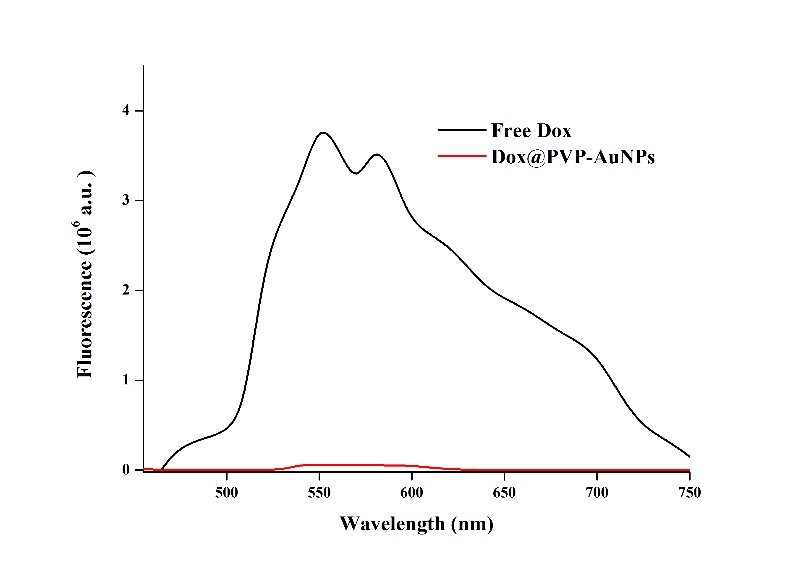 | b)  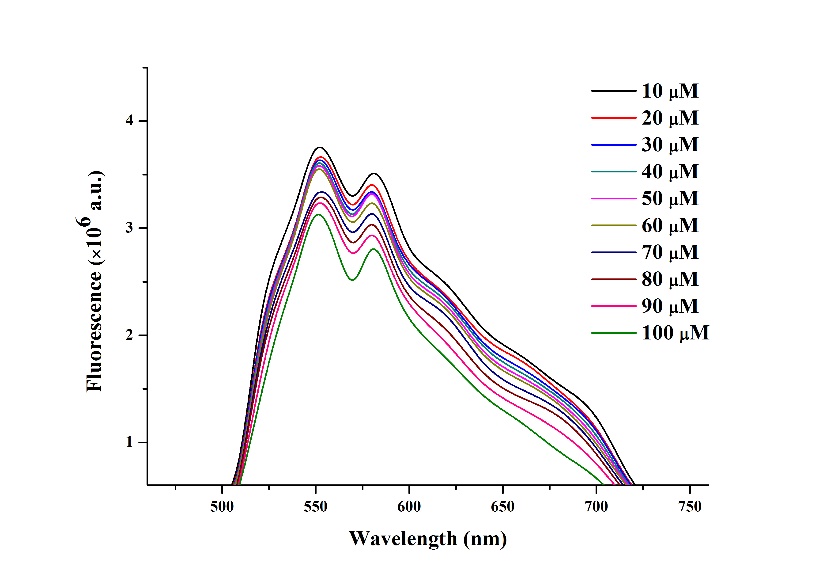 |
| --- | --- |
| c)  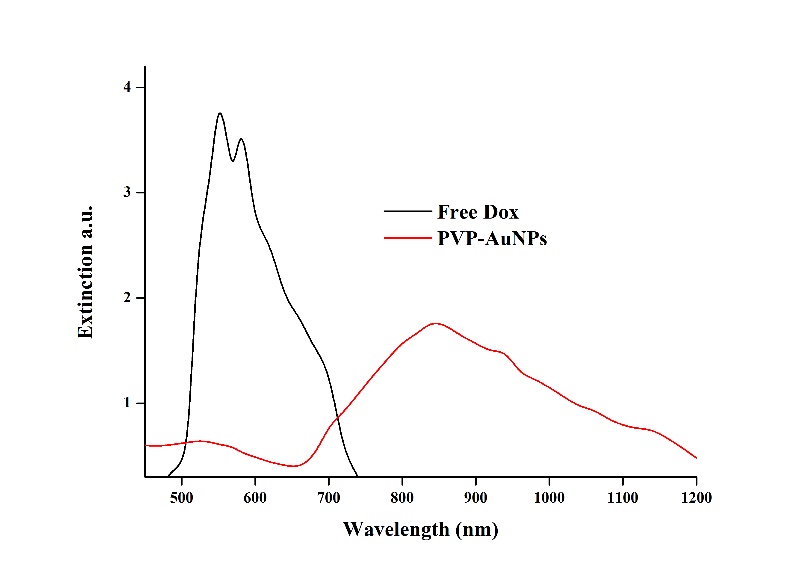 | d)  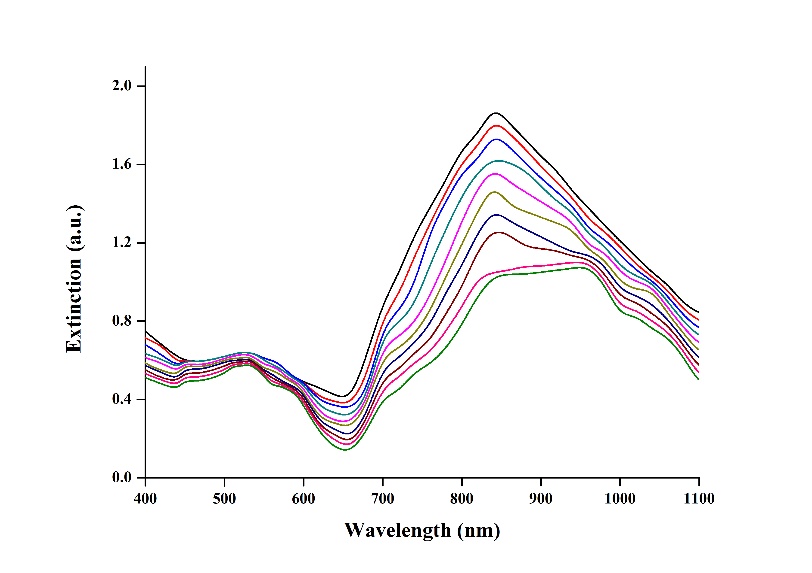 |

**Fig. S4** Colony formation of A549, H460 and H520 cells after 24 hr treatment with PVP-AuNPs, Free Dox and Dox@PVP-AuNPs was analysed using Crystal violet staining assay.

|  | **Control** | **PVP-AuNPs** | **Free Dox** | **Dox@PVP-AuNPs** |
| --- | --- | --- | --- | --- |
| **A549** | 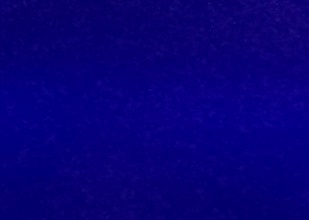 | 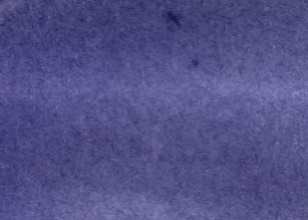 | 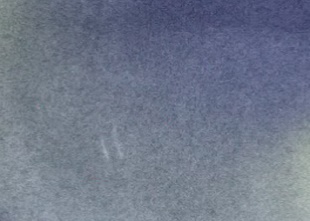 | 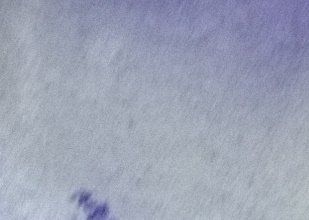 |
| **H460** | 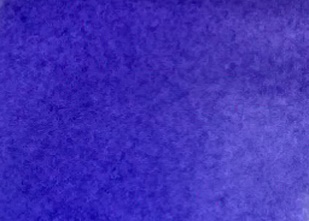 | 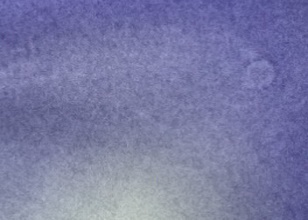 | 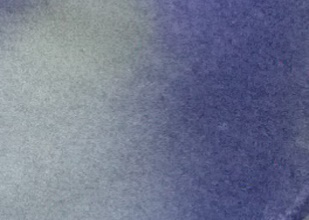 | 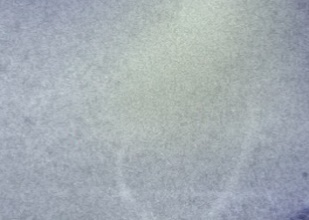 |
| **H520** | 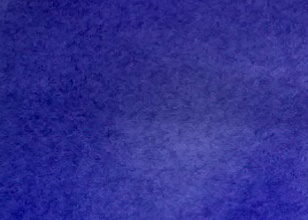 | 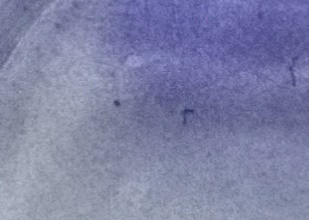 | 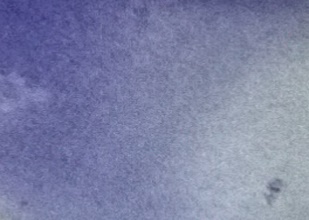 | 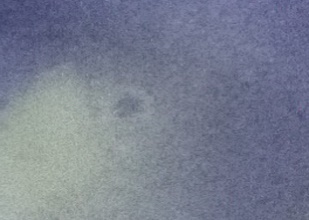 |

**Fig. S5** Morphology of control, PVP-AuNPs, Free Dox and Dox@PVP-AuNPs treated A549, H460 and H520 cancer cells were observed using a phase contrast microscope.

|  | **Control** | **PVP-AuNPs** | **Free Dox** | **Dox@PVP-AuNPs** |
| --- | --- | --- | --- | --- |
| **A549** | 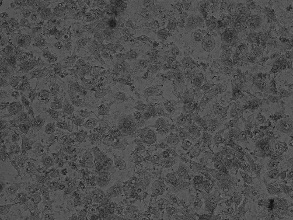 | 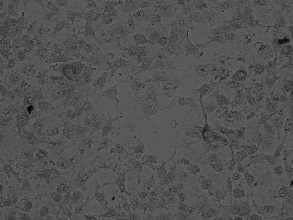 | 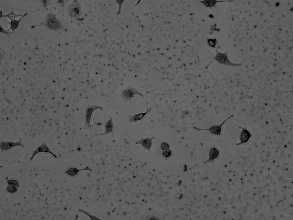 | 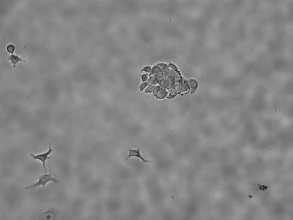 |
| **H460** | 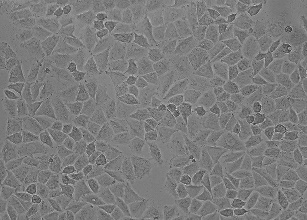 | 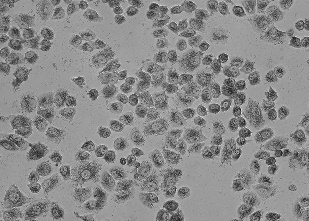 | 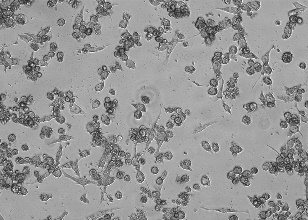 | 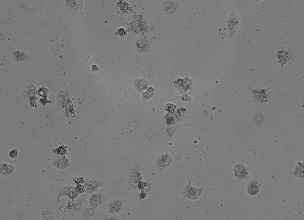 |
| **H520** | 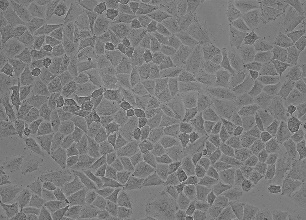 | 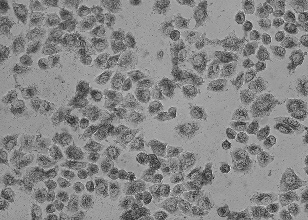 | 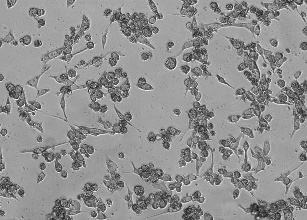 | 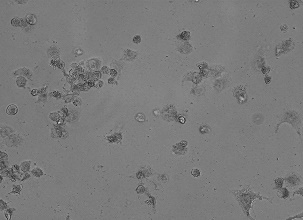 |

**Fig. S6** Fluorescent images of acridine orange and ethidium bromide stained lung cancer cells showing the induction of apoptosis following a 24 hr treatment with PVP-AuNPs, Free Dox and Dox@PVP-AuNPs.

|  | **Control** | **PVP-AuNPs** | **Free Dox** | **Dox@PVP-AuNPs** |
| --- | --- | --- | --- | --- |
| **A549** | 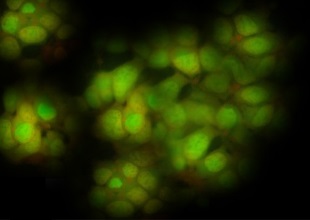 | 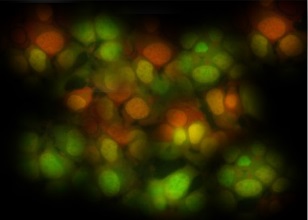 | 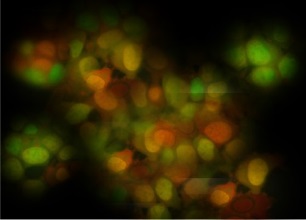 | 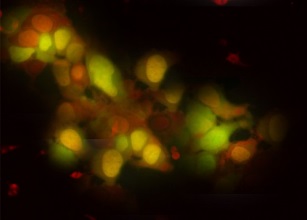 |
| **H460** | 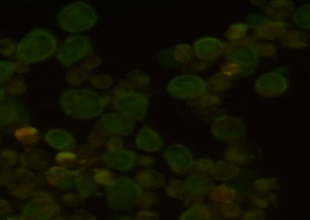 | 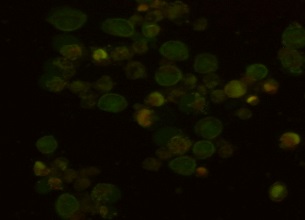 | 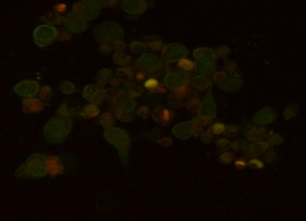 | 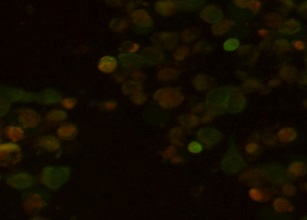 |
| **H520** | 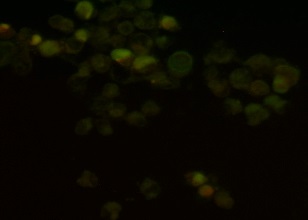 | 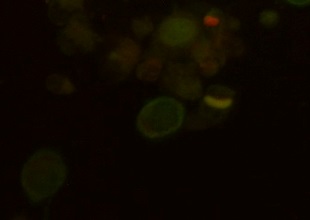 | 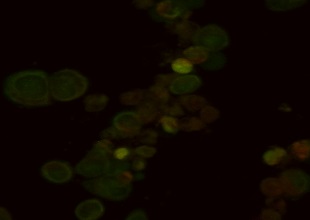 | 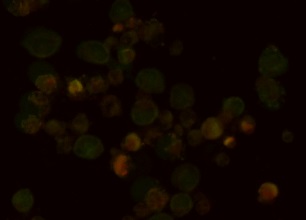 |

**Fig. S7** Representative images shows the morphological changes of A549, H460 and H520 lung cancer cells detected with Hoechst 33342 staining. Cells were treated with IC_50_ concentration of PVP-AuNPs, Free Dox and Dox@PVP-AuNPs for 24 hr and observed under fluorescent microscope.

|  | **Control** | **PVP-AuNPs** | **Free Dox** | **Dox@PVP-AuNPs** |
| --- | --- | --- | --- | --- |
| **A549** | 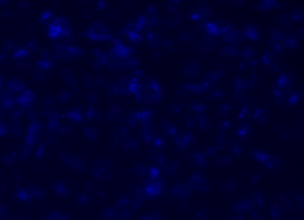 | 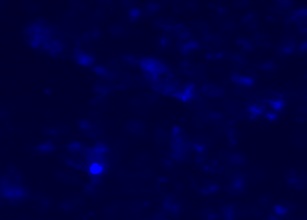 | 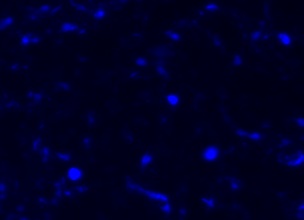 | 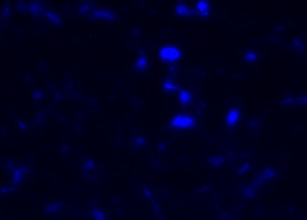 |
| **H460** | 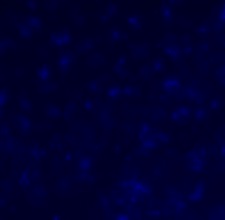 | 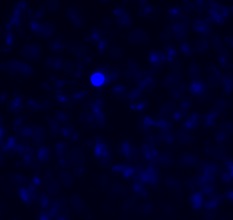 | 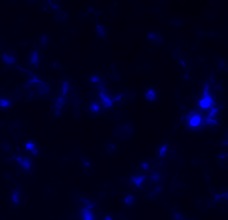 | 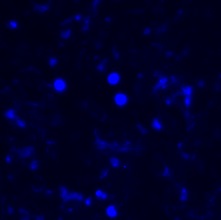 |
| **H520** | 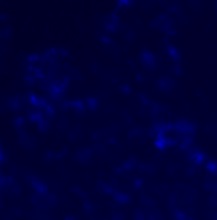 | 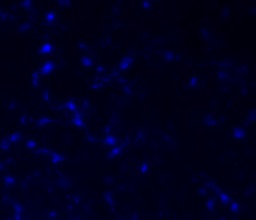 | 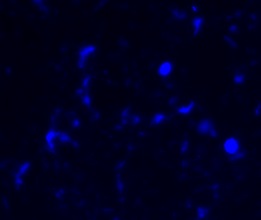 | 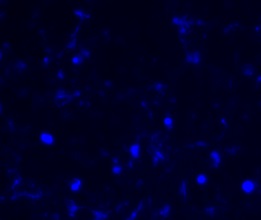 |

**Fig. S8** Generation of oxidative stress in lung cancer cells was monitored using DCFH-DA staining in Control (a), PVP-AuNPs (b), Free Dox (c) and Dox@PVP-AuNPs (d) treated cancer cells. Graph represents the fluorescence intensity of DCFH-DA stain measured by spectrofluorimetry in control and treated lung cancer cells.

|  | **Control** | **PVP-AuNPs** | **Free Dox** | **Dox@PVP-AuNPs** | **Fluorescence intensity** |
| --- | --- | --- | --- | --- | --- |
| **A549** | 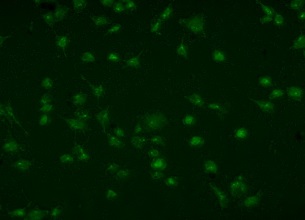 | 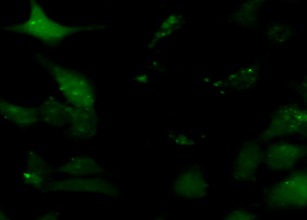 | 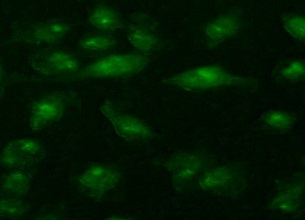 | 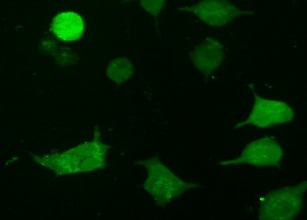 | 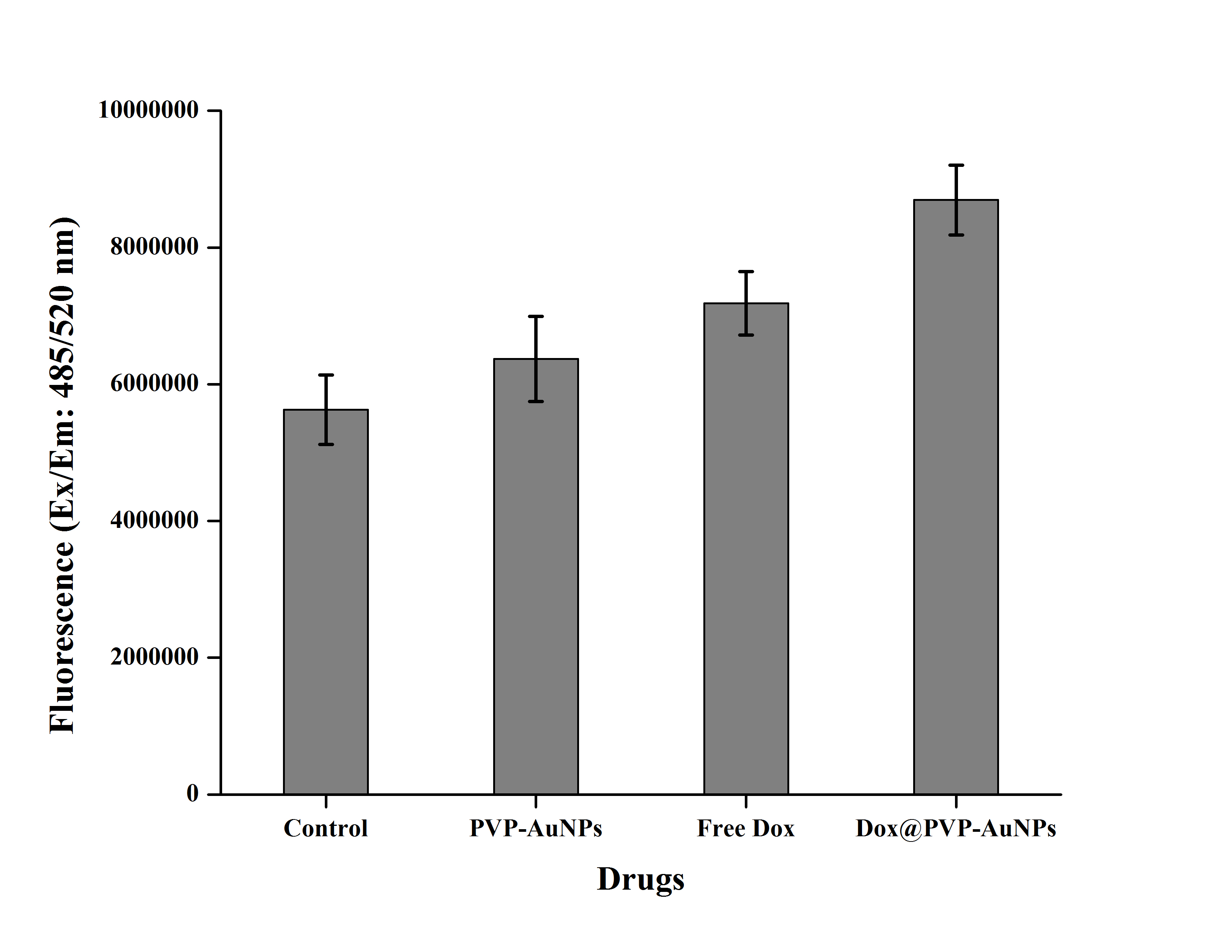 |
| **H460** | 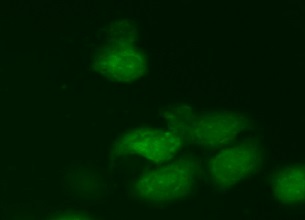 | 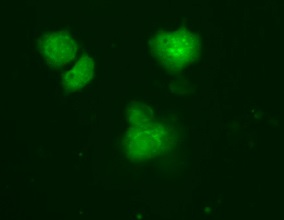 | 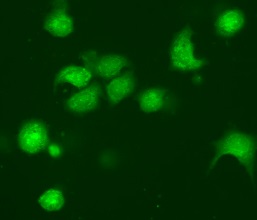 | 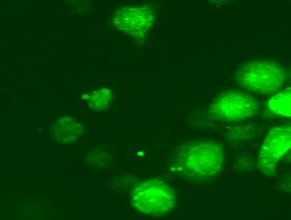 | 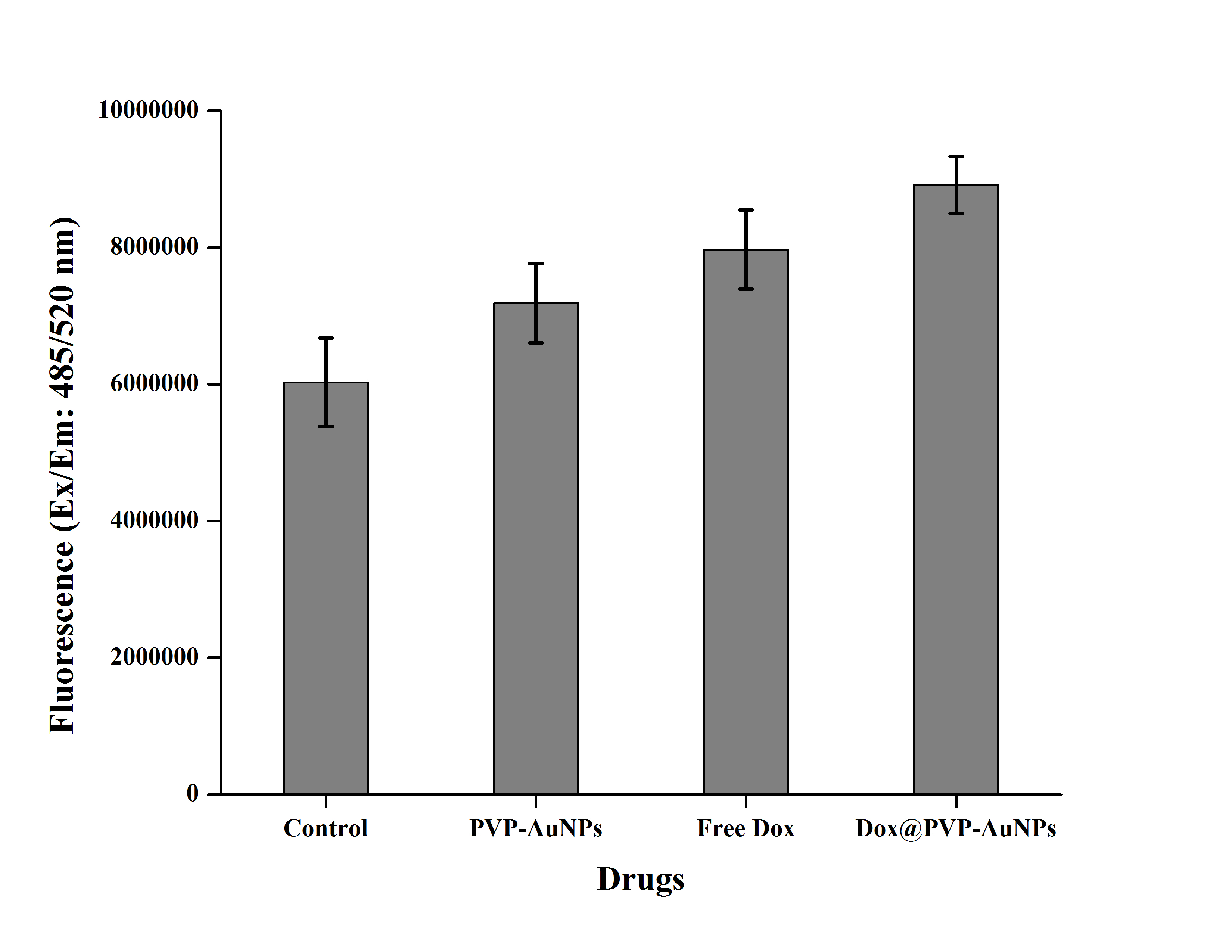 |
| **H520** | 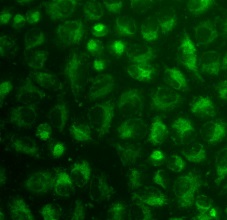 | 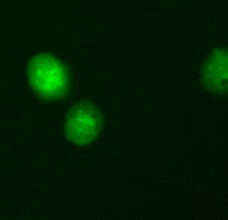 | 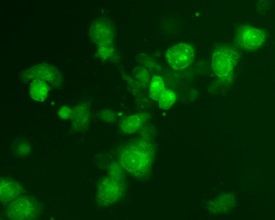 | 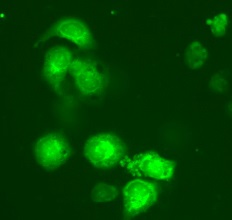 | 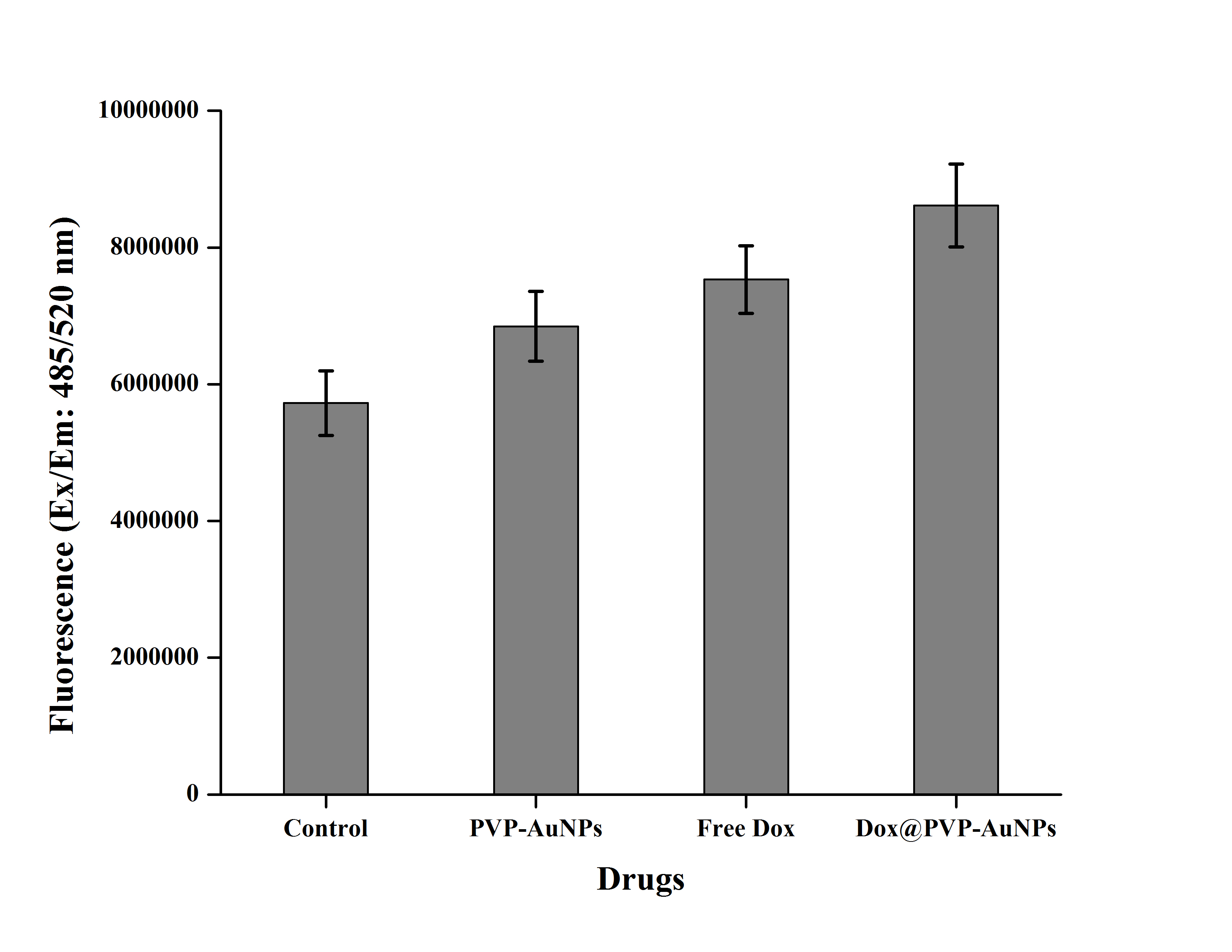 |

**Fig. S9** The sensitization of mitochondrial outer membrane during the treatment of control, PVP-AuNPs, Free Dox and Dox@PVP-AuNPs in A549, H460 and H520 lung cancer cells was detected by rhodamine 123 staining. Graph represents the fluorescence intensity of rhodamine 123 stain measured by spectrofluorimetry in control and treated lung cancer cells.

|  | **Control** | **PVP-AuNPs** | **Free Dox** | **Dox@PVP-AuNPs** | **Fluorescence intensity** |
| --- | --- | --- | --- | --- | --- |
| **A549** | 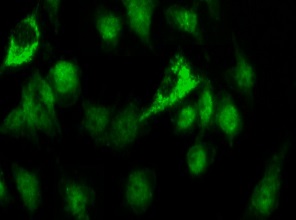 | 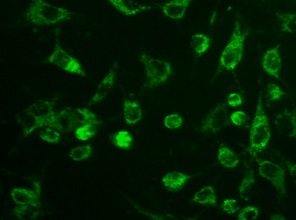 | 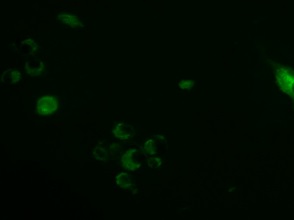 | 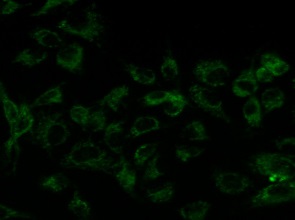 | 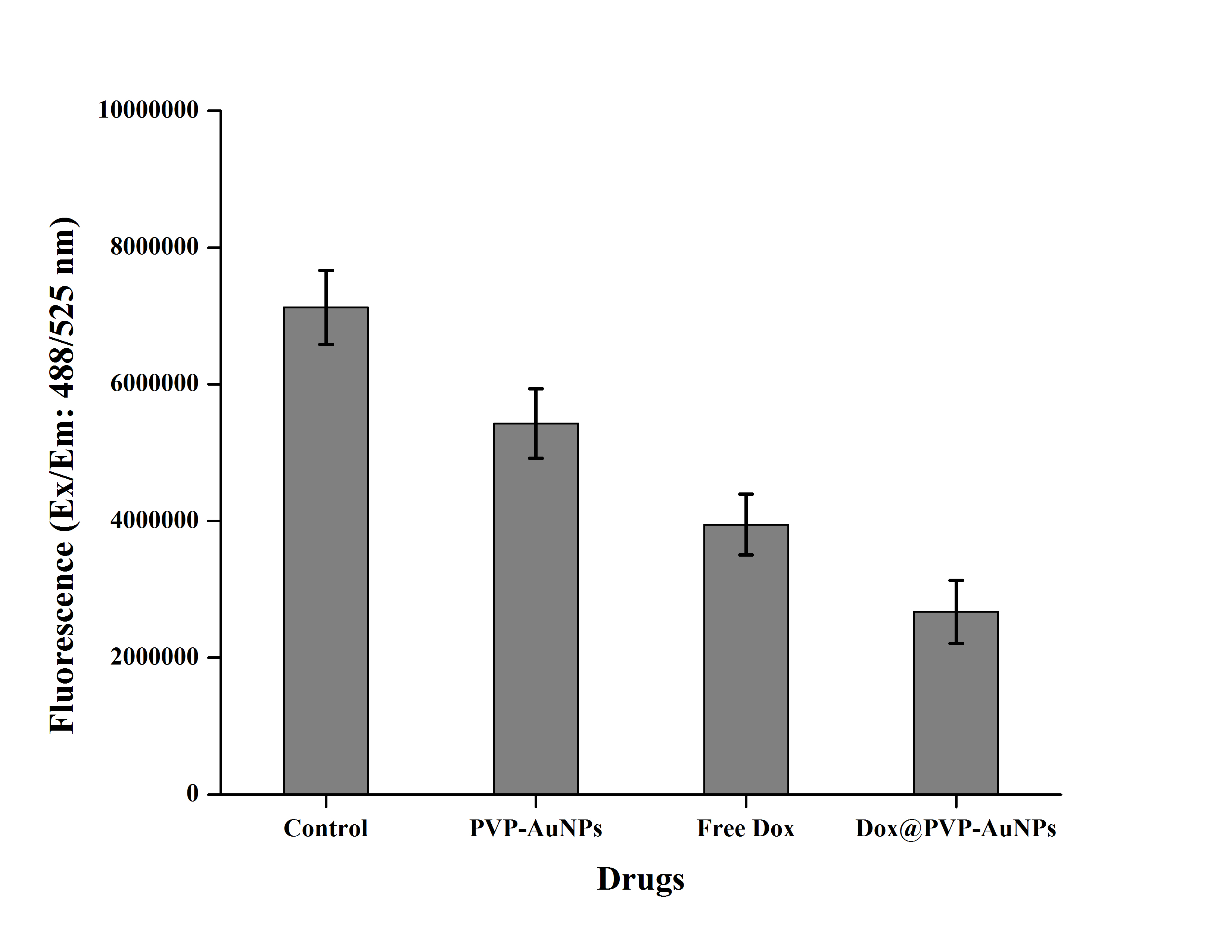 |
| **H460** | 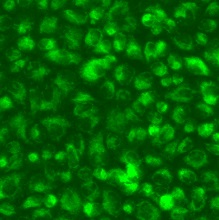 | 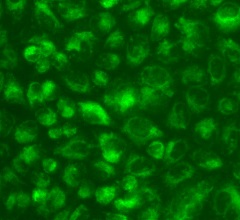 | 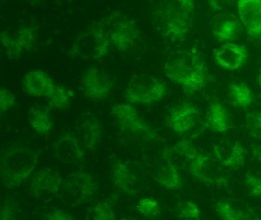 | 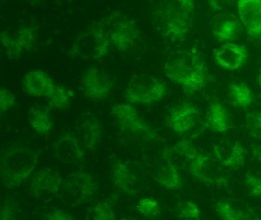 | 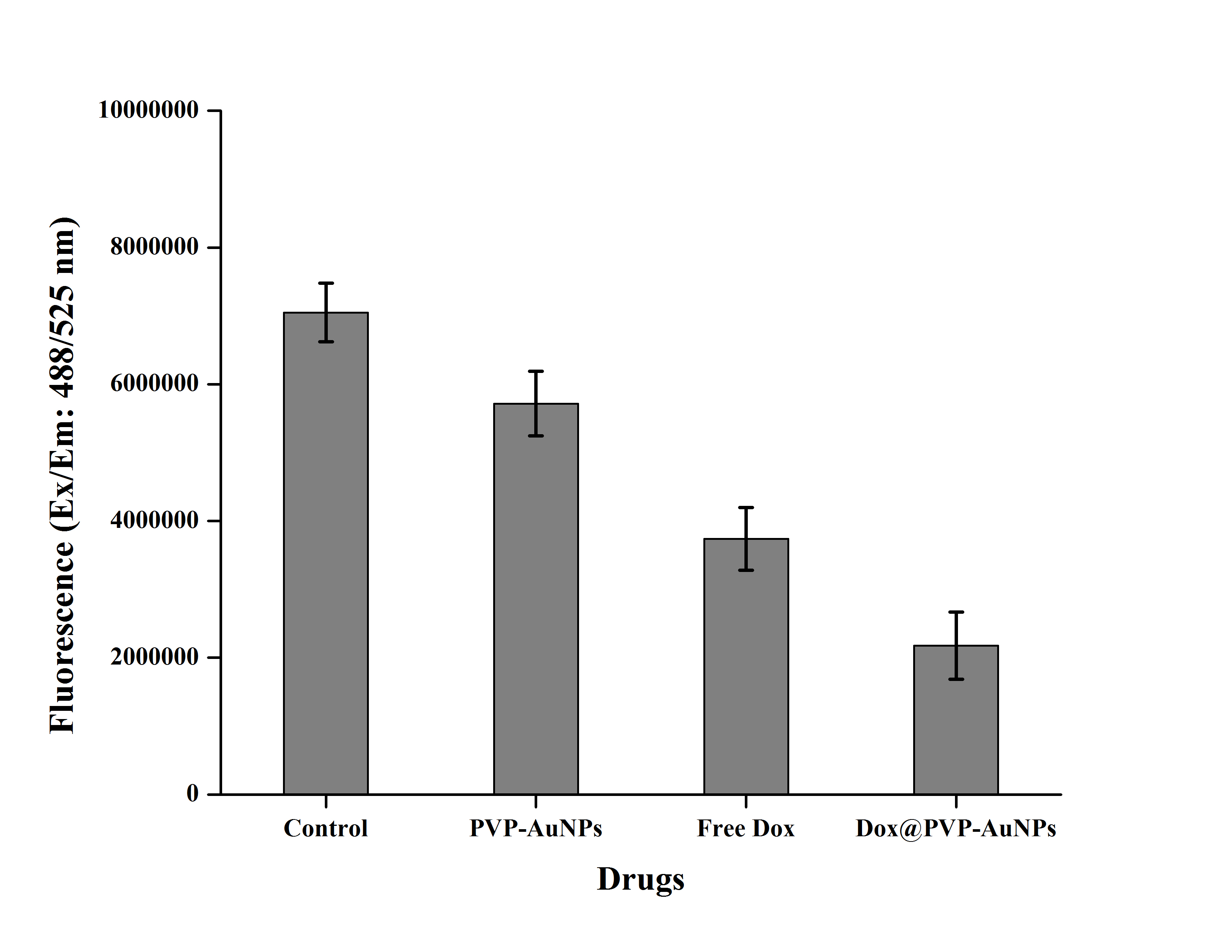 |
| **H520** | 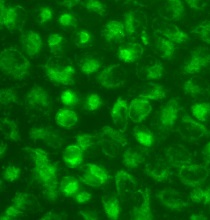 | 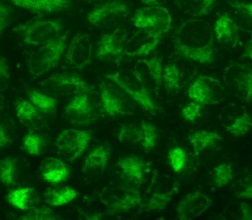 | 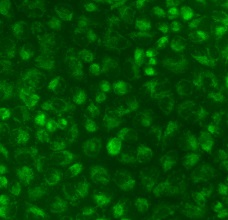 | 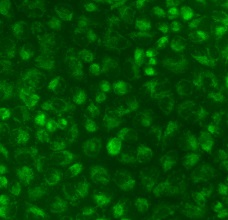 | 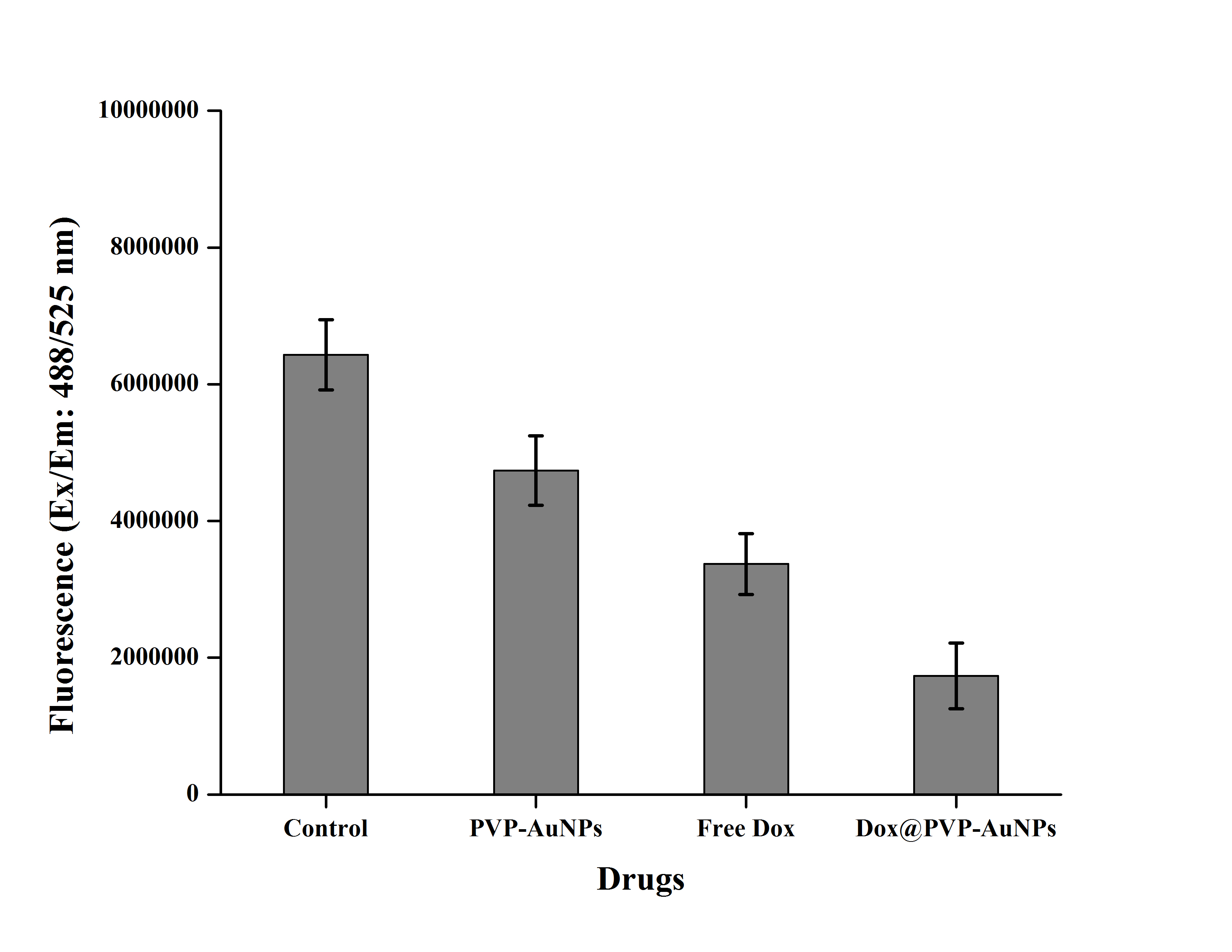 |

**Fig. S10** The significant increase of caspase 3 and caspase 9 in control, PVP-AuNPs, Free Dox and Dox@PVP-AuNPs treated A549 (a), H460 (b) and H520 (c) lung cancer cells.

| **a)**  **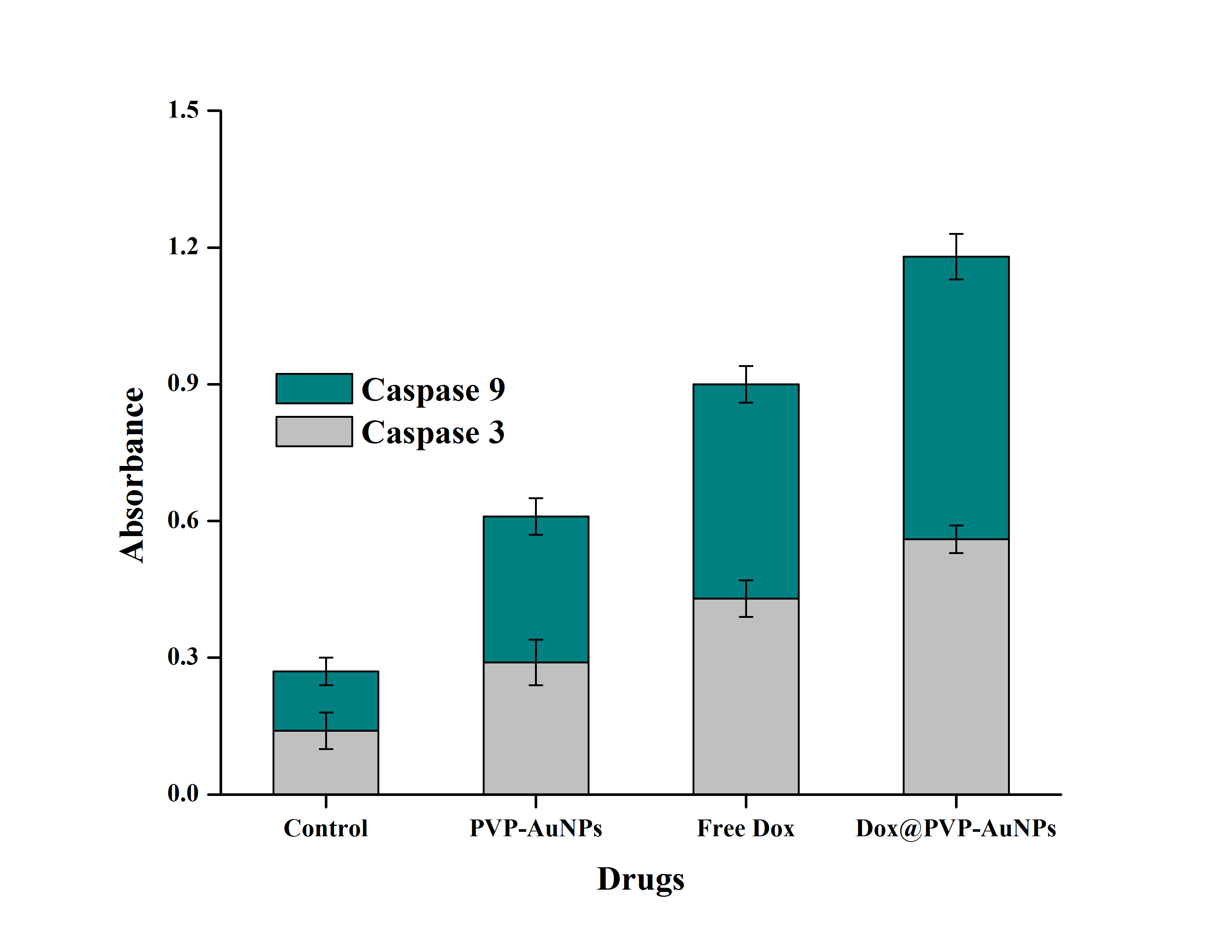** |
| --- |
| **b)**  **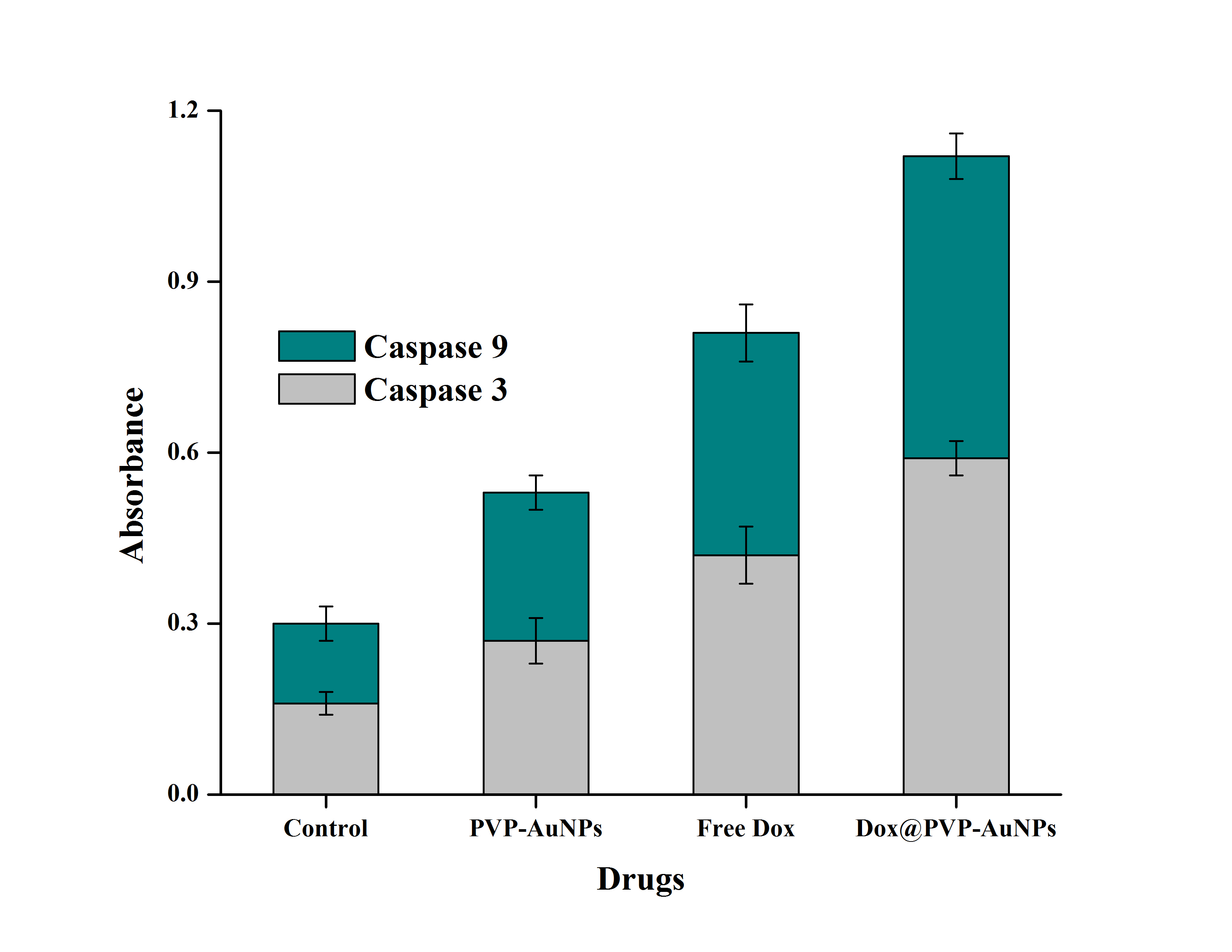** |
| **c)**  **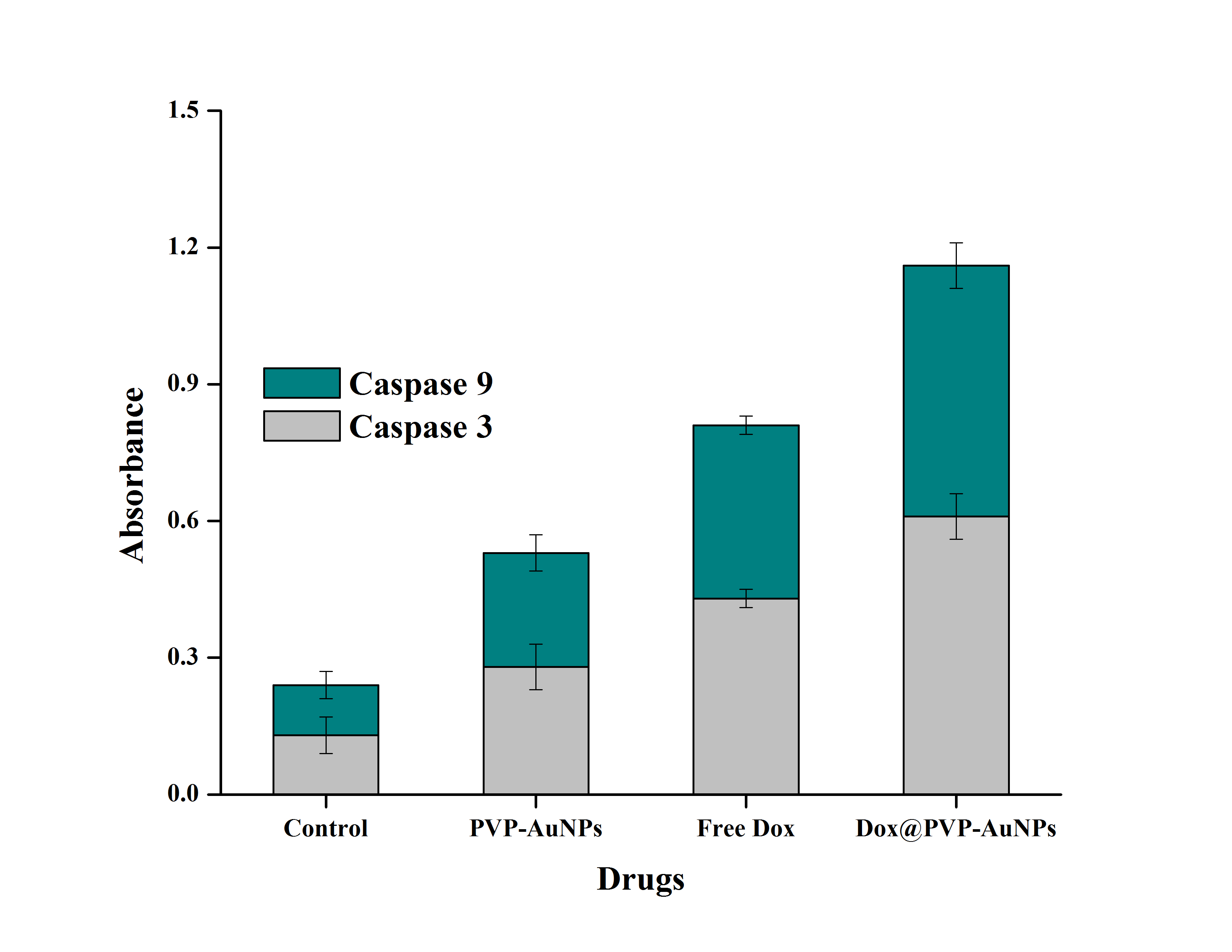** |

**Fig. S11** Schematic representation of synthesis of PVP stabilized AuNPs and conjugation of Doxorubicin with PVP-AuNPs.


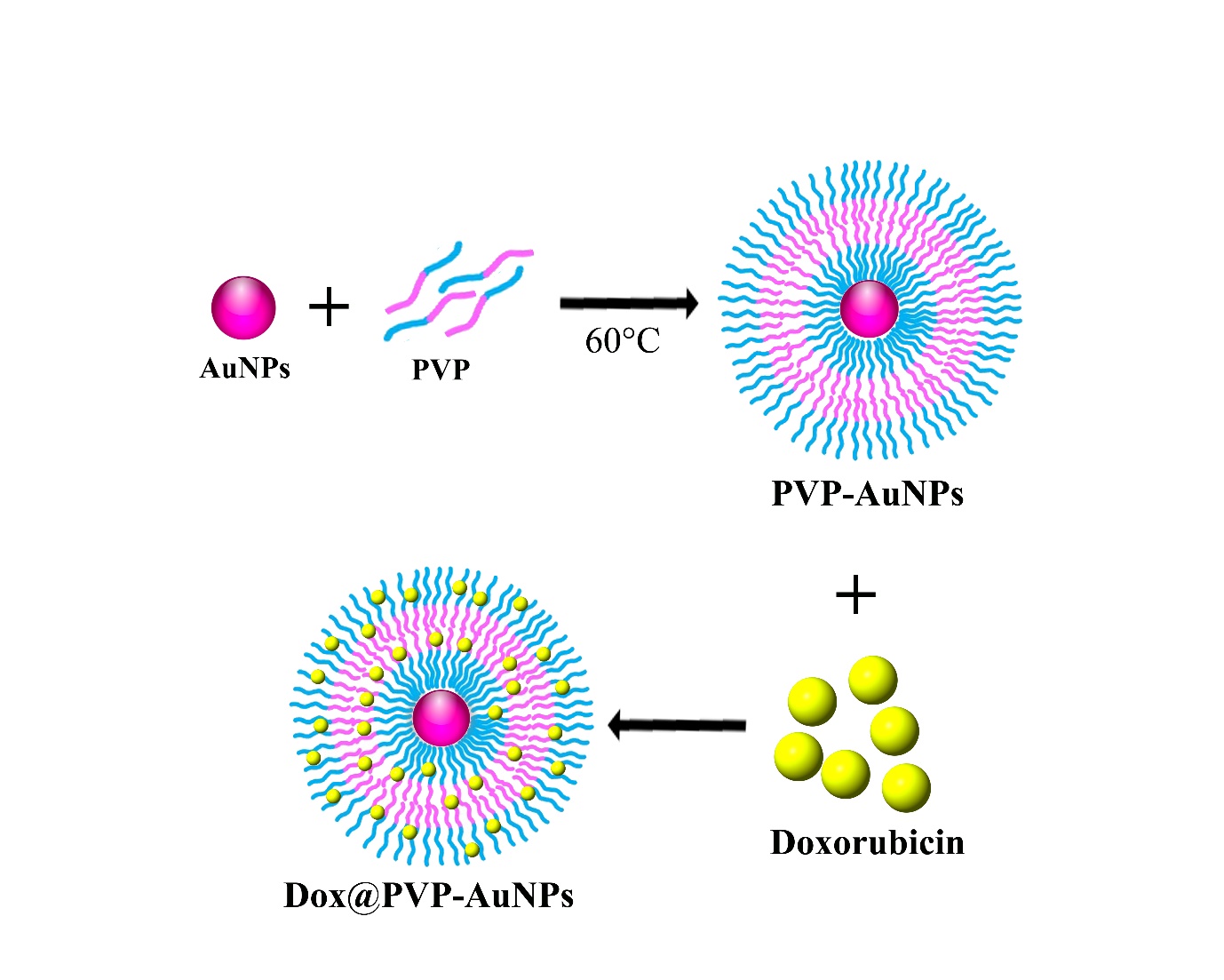


**Fig. S12 Schematic representation of Dox@PVP-AuNPs enhances intrinsic apoptosis in lung cancer cells.** Dox@PVP-AuNPs induces the oxidative stress in mitochondria which in turn activates the p53 tumour-suppressor protein. Activation of p53 initiates the intrinsic pathway by up-regulating Bax and Bak. Bax and Bak sensitize the outer mitochondrial membrane, resulting in release of cytochrome c, which binds to the adaptor Apaf-1 to recruit the initiator pro-caspase 9 into a signalling complex termed the apoptosome. Activated caspase 9 then cleaves and activates the effective caspase 3 to trigger apoptosis.


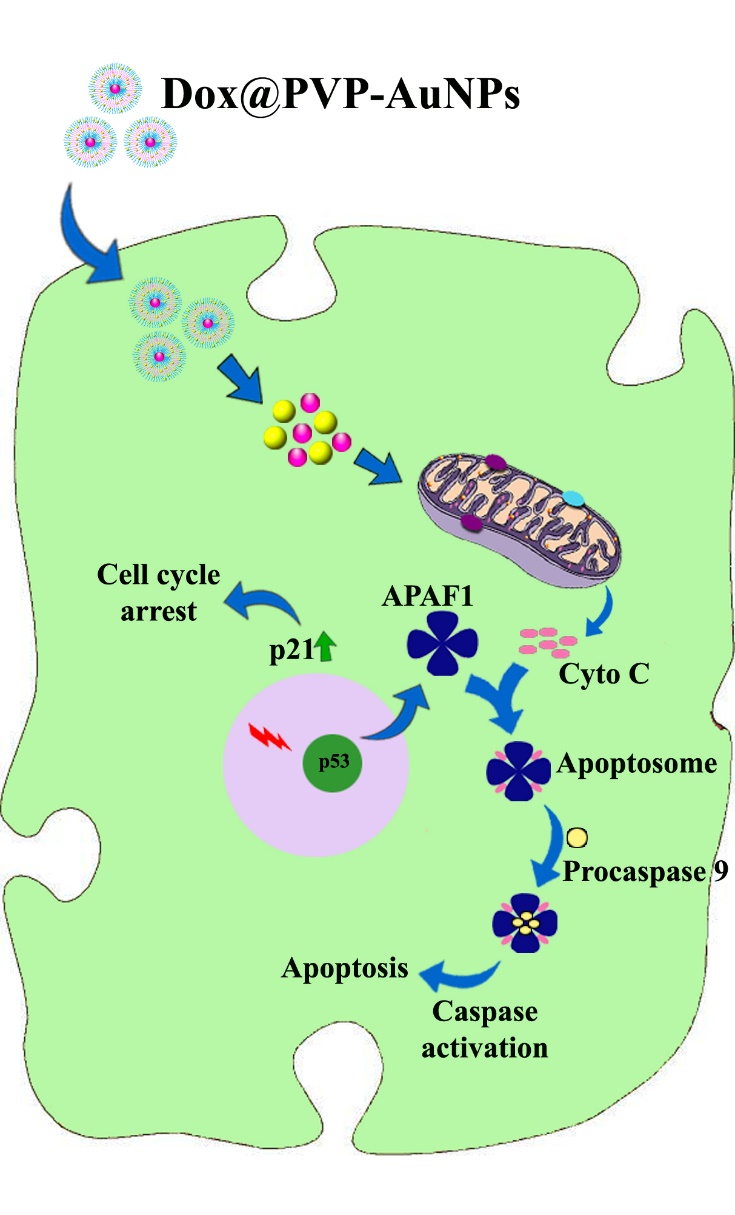


**Supplementary Fig. S13** shows the uncropped gels and the white boxes indicate the cropped regions for Fig. 5.

| **Fig. 5**  **GAPDH BAX**  **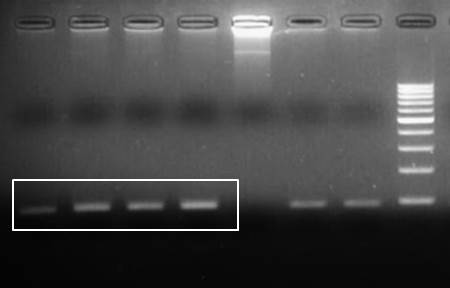**  **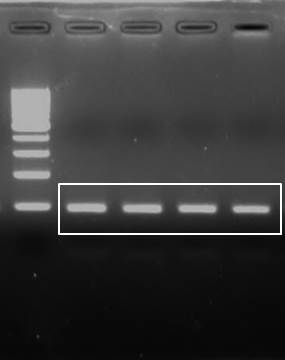**  **Cytochrome C**  **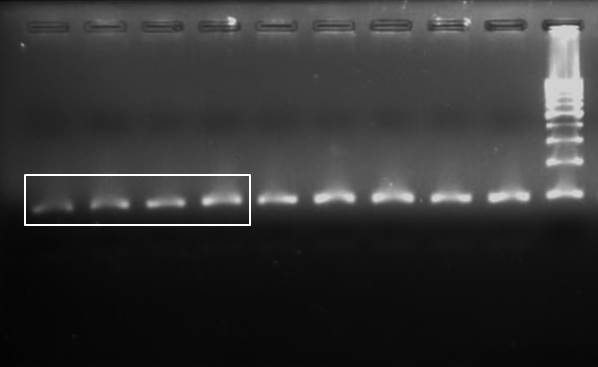** |
| --- |

**Supplementary Fig. S14** shows the uncropped gels and the white boxes indicate the cropped regions for Fig. 6.

| **Fig. 6**  **GAPDH p21**  **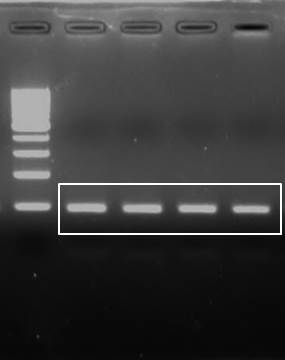 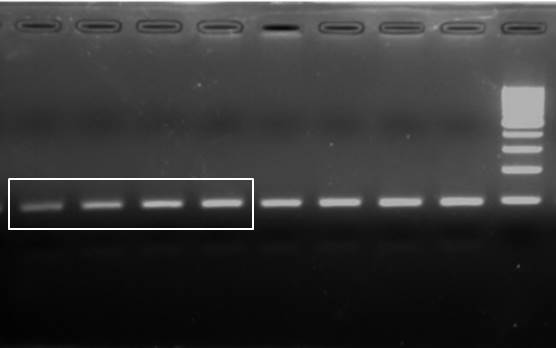**  **p53**  **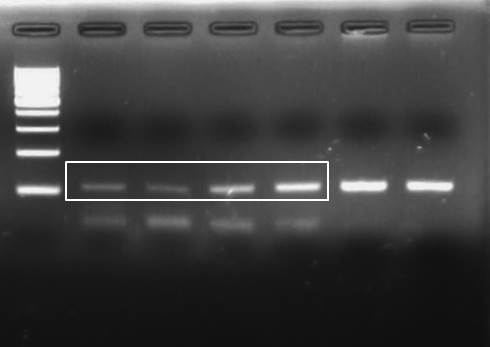** |
| --- |

**Supplementary Fig. S15** shows the uncropped gels and the white boxes indicate the cropped regions for Fig. 8.

| **Fig. 8**  **GAPDH Caspase 9**  **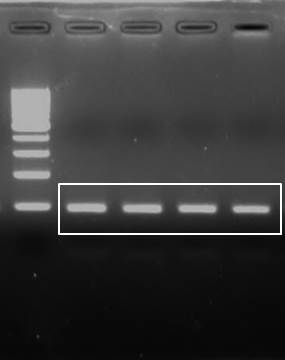**  **Caspase 3** |
| --- |
